# Differential expression proteomics to investigate responses and resistance to *Orobanche crenata *in *Medicago truncatula*

**DOI:** 10.1186/1471-2164-10-294

**Published:** 2009-07-03

**Authors:** Ma Ángeles Castillejo, Ana M Maldonado, Eliane Dumas-Gaudot, Mónica Fernández-Aparicio, Rafael Susín, Rubiales Diego, Jesús V Jorrín

**Affiliations:** 1 Institute for Sustainable Agriculture, CSIC, Alameda del Obispo s/n, Apdo. 4084, 14080 Córdoba, Spain; 2 Department of Biochemistry and Molecular Biology, University of Cordoba, Rabanales Campus, Córdoba, Spain; 3 UMR 1088 INRA/CNRS/UB (Plant-Microbe Environment) INRA-CMSE, BP 86510, 21065 DIJON Cedex, France

## Abstract

**Background:**

Parasitic angiosperm *Orobanche crenata *infection represents a major constraint for the cultivation of legumes worldwide. The level of protection achieved to date is either incomplete or ephemeral. Hence, an efficient control of the parasite requires a better understanding of its interaction and associated resistance mechanisms at molecular levels.

**Results:**

In order to study the plant response to this parasitic plant and the molecular basis of the resistance we have used a proteomic approach. The root proteome of two accessions of the model legume *Medicago truncatula *displaying differences in their resistance phenotype, in control as well as in inoculated plants, over two time points (21 and 25 days post infection), has been compared. We report quantitative as well as qualitative differences in the 2-DE maps between early- (SA 27774) and late-resistant (SA 4087) genotypes after Coomassie and silver-staining: 69 differential spots were observed between non-inoculated genotypes, and 42 and 25 spots for SA 4087 and SA 27774 non-inoculated and inoculated plants, respectively. In all, 49 differential spots were identified by peptide mass fingerprinting (PMF) following MALDI-TOF/TOF mass spectrometry. Many of the proteins showing significant differences between genotypes and after parasitic infection belong to the functional category of defense and stress-related proteins. A number of spots correspond to proteins with the same function, and might represent members of a multigenic family or post-transcriptional forms of the same protein.

**Conclusion:**

The results obtained suggest the existence of a generic defense mechanism operating during the early stages of infection and differing in both genotypes. The faster response to the infection observed in the SA 27774 genotype might be due to the action of proteins targeted against key elements needed for the parasite's successful infection, such as protease inhibitors. Our data are discussed and compared with those previously obtained with pea [1] and transcriptomic analysis of other plant-pathogen and plant-parasitic plant systems.

## Background

Broomrapes (*Orobanche *spp.) are obligate root parasites causing significant yield losses in many important crops [2,3]. Specifically, crenata broomrape (*Orobanche crenata*) is considered to be the major constraint for legume crops in Mediterranean countries [4]. The best long-term strategy for limiting damage caused by *O. crenata *is the development of resistant crops, but only moderate to low levels of incomplete resistance with a complex inheritance has been identified in crop legumes so far. This has made selection for resistance more difficult and has slowed down the breeding process. The quantitative resistance resulting from tedious selection procedures has resulted in the release of faba bean cultivars with useful levels of incomplete resistance, but this has not yet been achieved for pea or lentil cultivars [4,5]. In order to obtain long-term effective resistance, several resistance elements should be combined in one cultivar, and, consequently, detailed knowledge of legume-*O. crenata *interaction and of the mechanisms underlying resistance are prerequisites.

The *Orobanche *biological cycle comprises well-defined steps. Upon germination, stimulated by specific root host-exuded chemical signals, broomrape seed develops a small radicle that attaches itself to the host root and differentiates into a haustorium, the infective organ. After host tissue penetration and connection to the vascular system, the parasite begins to use the host resources, gradually forming a nodule or tubercle, from which a shoot arises and emerges from the soil to flower and produce seeds [2,6]. Successful parasite establishment creates a strong sink of nutrients and phothosyntates to the detriment of the host [3].

Several resistance and prevention mechanisms have been identified, one of the first lines of defense being the failure of host roots to stimulate *Orobanche *seed germination [3] and a number of studies have focused on identifying the host signals that induce germination and appressorium formation [7-9]. Subsequent resistance mechanisms will act by blocking host tissue penetration and connection to the vascular system. Among these are the typical plant mechanisms of defense against pathogenic microorganisms, such as the induction of pathogenesis-related (PR) proteins, peroxidases and phytoalexin biosynthetic enzymes, callose deposition and reactive oxygen species (ROS) accumulation [1,10-15]. Recent histological studies in legumes and sunflower have revealed that the unsuccessful infection of *Orobanche *is the result of the coordinate activation of several defense mechanisms during the early stages of the infection process. A physical barrier prevents the parasite from penetrating the host tissues, by lignification of the host endodermis [16], and cell wall strengthening by suberization, cross-linking and callose deposition [15,17]. Simultaneously, the production and excretion of phytoalexins [13,17] and occlusion of host xylem vessels by deposition of mucilage [16,18] will cause the necrosis and death of the parasite tubercles before their emergence.

The application of postgenomic tools has already provided significant clues to enhance our understanding of plant responses to abiotic stresses, pathogen attack or symbiotic interactions [19-23]. Gene expression changes are being monitored in various systems either by macroarrays, microarrays or subtractive suppression hybridization [19,24,25]. We have initiated a research project aimed at studying *O. crenata *interactions in legumes using a proteomic approach. In a previous work we compared the root proteome of two pea accessions differing in their susceptibility to *O. crenata *and reported the presence of higher levels of defense- and stress-related proteins further induced upon infection [1]. Furthermore, the decrease in proteins of the carbohydrate metabolism upon inoculation of the susceptible genotype reflects the reorganization of metabolic fluxes in the infected tissues as a consequence of the sink effect of the parasite on the host plant. This study highlighted the usefulness of this approach by providing the first clues to the plant's response to the parasite and its resistance at the protein levels. Nevertheless, the scant protein sequence information available for this crop in databases complicates the analysis and prevents a more comprehensive one of the proteome changes induced. Accordingly, we have chosen *Medicago truncatula *as a more tractable biological system to study changes in the legume root proteome in response to *O. crenata*. This system will provide excellent opportunities to compare our results with the information generated from a number of projects directed at establishing the root proteome of *M. truncatula *and the changes induced upon pathogen infection [26,27] and symbiosis [28-30], ; .

To this end, based on recent investigations carried out to assess the relative response of a collection of *M. truncatula *accessions to *Orobanche *spp. [31,32], we selected two of those displaying the most extreme differential responses following *O. crenata *infection: SA 27744, displaying early resistance, the parasite being halted at initial stages of the infection, and SA 4087, the genotype with the highest levels of infection ever reported in this species, but which is still quite resistant compared to susceptible pea, lentil or faba bean genotypes due to late-resistance, in which tubercles are formed and later become necrotic, preventing the emergence of most flowering shoots [31,33]. Here, we present the subsequent comparative proteomic profiling carried out to link the differential responses to *O. crenata *observed to differences detected in root protein expression. Root proteins from control and parasitized *M. truncatula *SA 27774 and SA 4087 plants were visualized by 2-DE and subsequent gel staining with Coomassie and silver nitrate. We report changes in the 2-DE map between both genotypes as well as in response to parasite infection. Some of the differentially present proteins were identified by MALDI-TOF/TOF analyses, and many of them belonged to the functional category of defense and stress-related proteins. The data presented in this paper suggest that resistance to broomrape in *M. truncatula *could rely on both a battery of general plant defense responses against different stresses and some more specific responses. In addition, the comparison of the present data with previous proteomic data obtained in pea [1] and in other systems allows a discussion about the common and specific responses of species.

## Results

### *O. crenata *seed germination, attachment and nodule formation

First, we conducted a Petri dish inoculation bioassay with both genotypes, SA 27774 (early resistance) and SA 4087 (late resistance) [31] in order to define the infection process stages at which each host prevented parasitism and to obtain a precise description of the defense response timing (additional file 1, Table 1). Germination of *O. crenata *seeds started to be visible 14 days after inoculation, once the conditioning period of ten days required for germination was completed. Twenty-one days after inoculation, the average percentage of germinated seeds was similar for SA 27774 and SA 4087 plants (44 and 48%) (additional file 1, A–B, Table 1). At this time, only 2.2 and 12.8% of the germinated seeds for SA 27774 and SA 4087, respectively, showed the radicle attached to the host root (additional file 1, A–D, Table 1). Nodules were only observed 25 days after inoculation in the SA 4087 genotype, the average number being 5.6 (additional file 1, F–G; Table 1).

**Table 1:** *Orobanche crenata *seed germination, attachment and nodule development on roots of *M. truncatula*^a^

Accession	% Germination^b^	% Attachments^c^	Number of nodules^d ^per plant
SA 27774	44	2.2*	0*
**SA 4087**	**48**	**12.8**	5.6

^a ^As determined by using the Petri dish bioassay [89]. Values are mean of five independent replicates.

^b ^Percentage of germination of *O. crenata *seeds that were at 0–3 mm from host root

^c ^Percentage of *O. crenata *germinated seeds that attach on *M. truncatula *roots.

^d ^Number of resulting nodules counted 25 days after inoculation.

* indicates that differences are statistically significant (LSD, P < 0.05)

In order to identify resistance mechanisms at critical stages of the parasitization process, we analyzed the root proteome of these *M. truncatula *genotypes in response to *O. crenata *21 dpi (complete resistance acting at early penetration stages in SA 27774) and 25 dpi (incomplete late acting resistance mediated by necrosis of parasite tubercle in SA 4087).

### Two-dimensional gel electrophoresis, mass spectrometry analysis and protein identification

Proteins were extracted from healthy- and inoculated- roots of SA 27774 and SA 4087 plants 21 and 25 dpi and resolved on 2-DE gels as previously described [1]. For each of the conditions analyzed (genotypes, treatments and sampling times), three replicates corresponding to independent protein extracts (biological replicates) were done. Additionally, for each protein extract, two 2-DE gels were performed, one to be stained with Coomassie and a second one with silver. Table 2 summarizes the amount of protein applied, number of spots resolved, differences observed, and number of proteins identified for each dye procedure used.

**Table 2:** Summary of the features of two-dimensional experiment

**Feature**	**Staining**	
	
	**Coomassie**	**Silver**
Protein/gel	500 μg	100 μg
Total spots resolved	400	800
Spot showing significant differences:		
- Total^a^	60 (15%)	76 (9.5%)
- Between genotypes	25	44
- Between control *vs*. inoculated SA4087 plants	20	22
- Between control *vs*. inoculated SA27774 plants	15	10
Total identified spots	35	14
Valid identified spots^b^	26	14
Unique proteins^c^	26	13

^a ^31 differential spots were observed with both staining methods

^b ^Spots identified with reasonable values for score, % coverage, number of matched peptides, and observed *Mr *and p*I *values close to theoretical data.

^c ^Number of different proteins identified.

The protein profile was highly reproducible among replicates from the same genotype/treatment/sampling time. Thus, the number of spots present in all replicates (expressed as percentage of common spots between replicates) was between 78–94% and 80–85% for intra-replicates of silver and Coomassie gels, respectively, and between 80–91% and 82–90% for the condition studied of silver and Coomassie gels, respectively, the majority of spots being within the 4–7 pH and 20–60 kDa *M*_*r *_ranges. The analysis across the three replicates of 2-DE maps of the two genotype (SA 4087 *vs*. SA 27774), treatments (control *vs*. inoculated), and sampling times (21 *vs*. 25 dpi) reveals qualitative (presence/absence) as well as quantitative differences (see additional material: files 2, 3, 4, 5, 6, 7, 8, 9, 10, 11, 12, 13).

**Figure 1:**
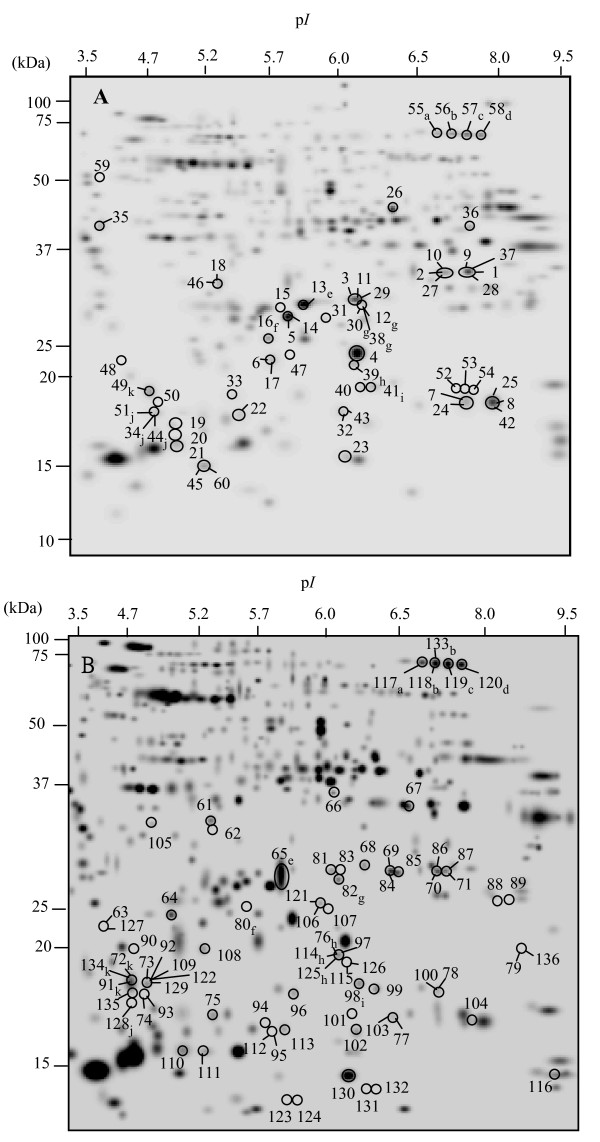
**Virtual 2-DE images from Coomassie (A)- and silver-stained (B) gels of *M. truncatula *root tissues**. Circled and numbered spots correspond to those showing changes between genotypes or treatments (additional files 2, 3, 4, 5, 6, 7, 8, 9, 10, 11, 12, 13). Spots assigned to more than one number, correspond to differences observed in more than one of the conditions compared. Molecular mass (on the left) and p*I *(on the top) were calculated using the PD-Quest software and standard molecular weight markers. Spots commonly observed in both staining methods display a subscript lowercase letter: the same letter in the virtual 2-DE image from Coomassie- and silver-stained gels mean that they have the same relative mobility on the gels.

Spots showing a significant change in volume (P < 0.05, Student's test, additional files 8, 9, 10, 11, 12, 13; LSD test, additional file 14) were selected for further analyses by MALDI-TOF/TOF. Out of the 60 spot differences detected in the Coomassie-stained gels, 25 reflect variations between genotypes and 20 and 15 spots corresponded to changes in response to infection with *O. crenata *in SA 4087 and SA 27774 plants, respectively (additional files 2, 3, 4, 8, 9, 10). Out of the 76 differences detected in silver-stained gels, 44 spots differentiated genotypes, and 22 and 10 spots were found differentially represented between non-inoculated and inoculated SA 4087 and SA 27774 plants, respectively, (additional files 5, 6, 7, 11, 12, 13). Virtual 2-DE gels from silver and Coomassie stained gels of *M. truncatula *root tissues displaying the differentially represented spots for the different situations compared are shown in Figure 1. In all, 31 differential spots were observed in both staining methods (Figure 1). Depending on the dye used, the number of stained spots spanned between ca. 400 for Coomassie and ca. 800 for silver. Gel analysis revealed that most spots from Coomassie-stained gels corresponded to spots detected also with silver (Figure 1). Additionally, silver stained an extra set of spots not detected by Coomassie. This is in agreement with previous observations pointing towards the fact that the differences observed in 2-DE pattern, in terms of the number of spots, result mainly from differences in the sensitivity between both dyes [34]. On the other hand, the comparison of Coomassie-stained gels reveals clear differences not observed in silver-stained gels. This intriguing result is likely to be linked to the large differences in the dynamic range between both dyes [34]. Interestingly, some of the spots differentially expressed 21 dpi for each of the situations compared (genotypes and treatments) seemed to correspond to the same proteins showing changes at 25 dpi, as deduced from their similar p*I *and *Mr *values (additional files 2, 3, 4, 5, 6, 7, 8, 9, 10, 11, 12, 13), i.e. spots 106 and 34 differentially expressed at 21 dpi were found at 25 dpi as spots 121 and 44, respectively.

Out of the 136 differential spots analyzed from silver- and Coomassie-stained gels, 49 were identified by searching in the *M. truncatula *EST database and the SwissProt databases. The proteins identified are listed in additional file 15, including the number of peptides that hit the protein and the sequence coverage, as an indication of the confidence in their identification. In most cases, similar experimental and theoretical *M*_*r*_/p*I *values were obtained, except for those spots identified by homology with sequences from a different organism. The identity of these spots was further confirmed by peptide fragmentation and MS/MS analysis (additional file 15). Only 9 of the differential spots analyzed did not match all the confidence identity requirements, and are indicated in additional file 15. A number of proteins were represented by more than one spot with slightly different *Mr *and p*I *values, suggesting that these changes in the proteome can be attributed to post-transcriptional modification, different members of the same functional family (small shift in the p*I*), or degradation products (significant differences between theoretical and observed *Mr *values). For example, spots 52, 53 and 54 were identified as being different forms of proteinase inhibitor .TC102534, each displaying slight differences in p*I *and *Mr *values and could probably reflect some PTMs occurring *in vivo *or be the consequence of artefactual modifications such as deamidation in the proteins during sample preparation and processing. Furthermore, several spots identified in this work corresponded to proteins encoded by different members of a gene family, i.e. spots 63, 114, 127, 129 and 134, representing members of the functional family of trypsin inhibitors; and spots 1, 2, 27, 28, 39, 40 and 69 were identified as chitinases. The differential expression of genes belonging to the same family has already been shown for plant genes belonging to chitinase [35] and trypsin inhibitors [36].

### Protein differences between SA 27774 and SA 4087 genotypes

We selected a total of 69 spots differentiating genotypes (additional files 2, 5, 8, 11). It is interesting to note that for both lines the number of proteins showing variations was greater when comparing genotypes than upon parasite infection. 35 spots were only detected or observed in larger amounts in non-inoculated SA 4087 plants, while 34 were more abundant in SA 27774 plants. Quantitative data for differential protein spots classified in this group are presented in additional material (additional files 8, 11).

After PMF analysis, 22 spots could be identified (additional file 15), corresponding to 19 unique proteins. Among the identified spots most represented in SA 4087 plants were typical pathogen defense-related proteins: chitinase (TC106842, representing spots 1 and 2), thaumatin-like protein PR-5b (TC94274, representing spots 4 and 6), cysteine protease (Q9STA4), beta VI allergen (TC68012), one of the enzymes for phytoalexin production, chalcone-flavone isomerase (TC69564), aldehyde reductase (TC59970) functionally related to the detoxification of xenobiotic stress, cyclophilin (Q8VX73, corresponding to spots 7 and 8) and proteasome subunit alpha type-6 (O48551) involved in protein folding. Three proteins belonged to the transcription and translation category: translation initiation factor 5A-3 protein (P56335), reverse transcriptase-beet retrotransposon-related (Q2HTR5) and ribosomal protein small subunit 4 (Q6SEK7).

With regard to the proteins present in larger amounts in SA 27774 plants, several proteins were identified, some of them representing different members of the multigenic families described above. Among the defense-related proteins were: a trypsin inhibitor (TC69848), chitinase (TC68269), thaumatin-like, PR-related (TC59501), and disease-resistant protein (Q8H816). In this group, also identified were a glycine-rich protein (O22385) and proteins involved in protein folding and transcription: cyclophilin (MtD20401) and a putative retroelement pol polyprotein (Q9SK57). Finally, protein TC72846 of an unknown function was also most represented in roots of non-inoculated SA 27774 plants.

### Protein changes in response to *O. crenata *infection

*Orobanche *inoculation triggers significant alterations in the 2-DE protein profile of roots from both *M. truncatula *genotypes. Comparison of 2-DE gel images and quantitative data for the selected differential spots are presented in additional material (additional files 3, 4, 6, 7, 9, 10, 12, 13).

Notably, most of the changes triggered by parasitic infection correspond to an increase in proteins related to defense responses. Thus, following parasitic infection of SA 4087 plants, 15 out of the 42 differences observed could be identified as corresponding to 11 unique proteins. Among the proteins showing an increase in intensity in response to infection, four were involved in defense reactions: chitinase (TC106842), glutathione-S-transferase (TC59483), a well-known marker of stresses associated with the generation of ROS [37], the glycine-rich RNA-binding protein (TC59317), generally related to cell wall reinforcement and which has recently been involved in the response of plants to pathogens [38,39], and a different isoform of the trypsin inhibitor previously identified (TC62239). Two additional spots were most represented in inoculated SA 4087 plants, and were identified as fructose-bisphosphate aldolase (O65735), which catalyzes one of the reactions of the glycolytic/gluconeogenic pathways, and a guanine nucleotide-binding protein subunit beta-like protein (O24076) which is involved in a variety of cell processes including protein synthesis, protein and vesicle trafficking, cell differentiation and proliferation, and signal transduction.

Nine spots decreased in intensity upon inoculation in SA 4087 and corresponded to 8 proteins. Five of them are defense-related and belong to the gene families described above: chitinase (TC106842, spots 27, 28 and 40), and a glycoside hydrolase (Q2HU16), which has been previously described. A proteasome subunit alpha type 7 (Q9SXU1), and a TH65-like protein (Q949J3) involved in turnover and signaling processes and a kinesin motor protein (Q2QMU6) were also identified. Two hits, a TBP-binding protein (Q8H2U2, spot 33) and Q94LH93 (spot 29) must be considered with caution as they do not fulfill the requirements for identity confidence (additional file 15).

For the SA 27774 genotype, 12 of the differentially expressed spots after infection were identified as corresponding to 10 unique proteins. Three of the proteins detected in larger amounts in inoculated roots were identified as a proteinase inhibitor (.TC102534 spots 52, 53 and 54), three different members of the trypsin inhibitor functional family (TC69848, MtC00300 and TC69291), which have been previously involved in pathogenic and symbiotic interactions. Finally, a protein with an unknown function increased in inoculated plants (A2ZEG4).

A glycine-rich RNA binding protein (Q9SP10) was detected in smaller amounts in inoculated roots and, a protein with an unknown function was detected in non-inoculated plants (Q9SH68). Finally, another four proteins that changed in roots of SA 27774 plants after inoculation were identified but need to be treated with caution: a putative transaldolase (Q5JK10), OSJNBa0056L23.24 (Q7XL33), a copy-type reverse transcriptase-like protein (Q9M197) and a hypothetical protein (A2ZEG4).

### Protease inhibitor activity

**Figure 2:**
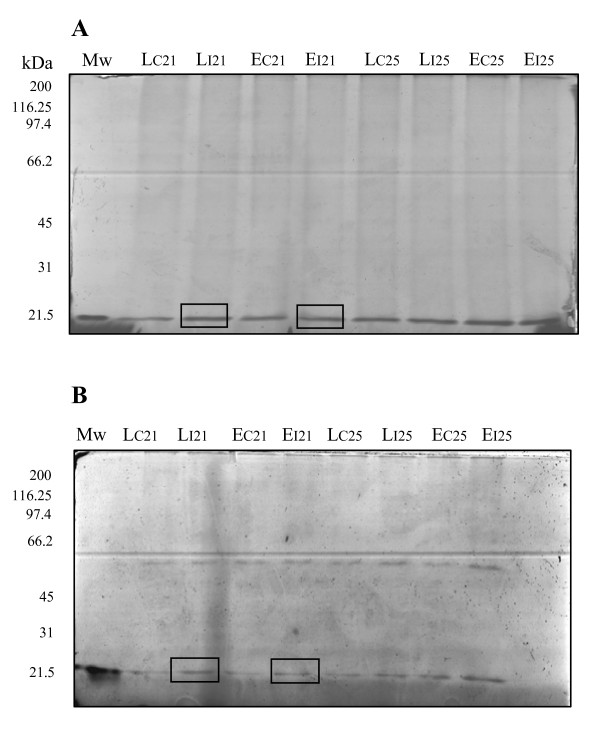
***Medicago truncatula *roots pattern after run in SDS-PAGE- bearing gelatine following by trypsin digestion**. A: samples heated for 3 min at 100°C in denaturing conditions, B: samples dissolved at room temperature in non-denaturing conditions. Squares indicate a moderate increase in the band intensity for both genotypes at 21 dpi, and in both situations (denaturing-A and non-denaturing-B conditions).

The identification of several spots as members of the trypsin inhibitor family, promoted us to investigate protease inhibitor activity during the interaction. For that, we used the method developed by Segarra et al. [40] and described in M&M to directly visualize protease activity in gel. Proteins from root tissue of both genotypes, and from control and infected plants were separated in 10% acrylamide SDS-PAGE slabs containing 0.1% gelatine. Gels were then incubated in the presence of trypsin (40 μg ml^-1) ^and, after Coomassie staining, blue stained bands revealed a protease inhibitor activity. When denaturing conditions were used (boiling the sample and using reducing agents) a unique 21 kDa band was observed (Figure 2A). However, two bands with different molecular weights (21 and 66 kDa) appeared when native conditions were used (Figure 2B). This makes sense considering that trypsin inhibitors have two subunits, as previously described in Segarra et al. [40]. Quantitative differences were observed for the 21 kDa band corresponding to the denatured small subunit, this being more intense in inoculated plants from both genotypes at 21 dpi.

## Discussion

In this work we gained a deeper insight into the molecular basis of legume resistance to parasitic plants by analyzing proteome changes in roots of two *M. truncatula *accessions in response to *O. crenata *infection. In order to obtain a more comprehensive analysis, we used accessions SA 27774 and SA 4087 halting *O. crenata *infection at different stages of the parasite cycle. In addition, considering that gel imaging analysis represents a compromise between the detection of minor spots and the limitation of saturated abundant spots, we used two different staining procedures, Coomassie and silver nitrate. The different sensitivity between both dyes, compatible with subsequent analysis by MS, led to the detection of a broad range of spots belonging to more abundant and less abundant proteins [34].

After gel image analysis across three replicates, those differences corresponding to proteins present in larger or smaller amounts between genotypes or in response to infection were selected for further MS analysis only if proven to be statistically significant, as determined by Student's test (P < 0.05).

With regard to protein identification, about 57% of identified proteins corresponded to *Medicago *specific matches (additional file 15). Only the legumes *Cicer arietinum *and *Glycine max *were close phylogenetically to *M. truncatula*, the others being species (*Arabidopsis thaliana*, *Oryza sativa*, *Ricinus communis *and *Solanum tuberosum*) more distantly related. For most spots, the values obtained for the standard parameters considered as being a proof of accurate identification when using MS, such as the number of peptides hitting the sequence of the homologous protein, score and percentage of sequence coverage together with the values obtained for experimental *Mr *and *pI *values, in good agreement with theoretical values, encourages confidence in their identification. This was not the case for identification based on homology with sequences from different species, which is considered uncertain and should therefore be interpreted with care [41,42]. These identifications were only considered to be reliable after a further MS/MS analysis of the fragmented peptides (additional file 15). Accordingly, those spots that are indicated in additional file 15 will not be discussed any further.

Concerning the success in their identification, it is interesting to mention that more proteins were identified from gels stained with Coomassie, although the number of protein changes detected using silver dye was higher (Table 2). This is consistent with the difference in the amount of proteins used for loading 2-DE gels depending on the dye used afterwards, much smaller for silver-stained gels (100 μg) and the different dynamic range for each staining procedures [34].

Proteins were assigned to functional categories (additional file 15) based on sequence homology or annotated function. These classifications are predictive and we have emphasized below if the protein function has been experimentally validated. From the functional clustering of the proteins identified for each of the situations, a clear trend emerges: most of the identified proteins are typical pathogenesis-related proteins induced during defense-responses against a broad range of microorganisms, or they belong to functional families involved in a number of stress- responses. Important intercellular communication processes, especially those associated with defense and development, occur in the plant apoplast. Secreted proteins (collectively called the secretome) are involved in pathogenic stresses, environmental stresses, cell-cell recognition, and development. To enter the secretory pathway via the endoplasmic reticulum (ER), it is necessary for secreted proteins to have an N-terminal signal sequence. Upon cleavage of the signal sequence, secreted proteins are normally transferred to the Golgi before being released into the extracellular space by vesicle fusion with the plasma membrane [43]. Some of the proteins identified in our work are predicted by SignalP to have an N-terminal signal sequence and thus to enter the ER-dependent secretion pathway (additional file 15). These are chitinase, thaumatin-like protein PR, trypsin inhibitor, beta VI allergen, glycoside hyrolase and glycine-rich RNA binding protein among others. These proteins have been previously found in secretome of *M. truncatula *and soybean plants [44-46,43], all of them being related to defense and signaling.

A smaller group of proteins was involved in metabolic functions, protein folding and turnover. Below, we discuss each functional group and the behavior pattern observed for the conditions studied (genotypes and response to infection).

### Defense-related proteins

Remarkably, the majority of proteins identified in this study were related to defense, with most of them being more represented in one of the two genotypes analyzed or upon parasite infection. Among these are: several trypsin inhibitors, proteases, chitinases, thaumatin-like PR-proteins, and glycine-rich RNA binding protein. Several cyclophilins, lately related to defense reactions in the *Arabidopsis*-*Pseudomonas *system, were also identified in the study and from more than one spot differing slightly in its p*I *value. This supported the case for the presence of different isoforms, and probably some PTMs, although alternative processing at the mRNA level cannot be excluded and could explain differences in *Mr*.

#### Trypsin inhibitors

**Figure 3:**
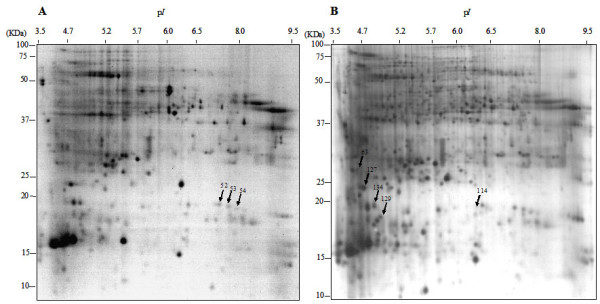
**2-DE from root extracts of SA 27774 *M. truncatula *genotype, Coomassie- (A) and silver- (B) stained**. Arrows indicate the position of protease inhibitor and trypsin inhibitor proteins.

A number of studies directed at studying global responses of plants at the mRNA and protein levels reveal that trypsin inhibitors are induced in response to a wide range of abiotic and biotic stresses, as well as during symbiotic interactions [47-49]. In addition, alteration in the expression of several trypsin inhibitors was reported in relation to insect resistance in different plant systems through their proteinase inhibitor activity, acting on key proteolytic proteins from the invading organisms [36,50,51]. We detected four proteins belonging to this functional group: TC69848, only visible in 2-DE gels from early resistant SA 27774 plants and further over-represented during response to parasite infection, MtC00300 and TC69291 increasing in these plants upon infection, and TC62239 increasing in roots of parasitized SA 4087 plants. This behavior is consistent with these proteins acting against *Orobanche *structural or metabolic components and stopping parasite development during the root penetration attempt. Notably, proteinase inhibitor .TC102534 was identified from three different spots displaying small differences in their p*I *values, probably corresponding to PTMs. The general trend for this group of proteins is to increase in roots of SA 27774 in response to *O. crenata *infection. The relative position of these proteins on a real gel is shown in Figure 3. These results are in good agreement with different members of the same gene family displaying differential expression patterns during a biological response. This has been observed for genes encoding chitinases and trypsin inhibitors during symbiotic and pathogenesis, respectively [35,36], and may argue in favor of the hypothesis that different members of this gene family play different roles in restricting *O. crenata *infection at different stages of the penetration process. It is well known that at early penetration stages *Orobanche spp*. secretes enzymes like peroxidases and pectin methylesterases [52,53]. This secretion may change the composition of host cell walls and middle lamellae, making them weaker and more vulnerable to the attack. Several isoforms of Kunitz trypsin inhibitors have been recently identified in xylem sap and apoplast proteome of *Glycine max*, being related with defense and signaling [45]. We can speculate that, in our system, different members of the protease inhibitor family present in resistant host plants actively responding to parasitisation, might target proteolytic enzymes secreted by the parasite to break through the physical barrier and reach the vascular cylinder, hence stopping parasite invasion. A recent study to monitor gene expression in the same biological system (*M. truncatula *accessions SA 27774 and SA 4087-*O.crenata*) has detected changes in the steady-state transcript levels of several trypsin inhibitors (Dita MA, Die J, Román B, Krajinski F, Kuster H, Moreno M, Cubero J, Rubiales D: Gene expression profiling of *Medicago truncatula *roots in response to the parasitic plant *Orobanche crenata*, submitted). These data further stress the implication of this functional group of enzymes in plant-parasitic plant interaction and resistance.

To validate experimentally the protein function, the trypsin inhibition activity of the samples was demonstrated by in-gel assay. On a SDS-PAGE zymograme, a band of 21 kDa was observed for all the samples tested. Furthermore, quantitative differences between control and inoculated plants of both genotypes were observed at 21 dpi (Figure 2A). These results are in agreement with those obtained from MALDI-TOF/TOF analysis, identifying three isoforms of trypsin inhibitor with a *Mr *of 21 kDa (TC62239, TC69848, MtC00300) in SA 4087 and SA 27774 inoculated plants. Another band appeared (66 kDa) under non-denaturing conditions (Figure 2B). This is consistent with previous data reported by Segarra et al. [40] in wheat leaves apoplast, the most active form being a 66 kDa oligomer. It is very likely that this native form of the trypsin inhibitor was not identified in our 2-DE analysis due to the denaturing conditions used.

#### Chitinases and Thaumatin-like protein, PR-5

Plant chitinases belong to a gene family whose products display different expression patterns in various *M. truncatula *tissues, and those correlate with different phenotypes upon a number of stresses, including symbiotic and pathogenic interactions [35,54,55]. Two chitinases have been found in both genotypes: TC68269 was observed in a greater amount in healthy SA 27774 root tissues, six isoforms of TC106842 were unique to SA 4087 roots and also decreased in intensity in response to *O. crenata*. To our knowledge, *O. crenata*, like other Angiosperms, does not contain chitin, and therefore, a specific role in resistance to *Orobanche *seems unlikely. Paradoxically, alterations in chitinases have also been detected and related to resistance in two other studies covering molecular aspects of plant-parasitic plant interactions: gene expression analysis conducted in *Arabidopsis thaliana *-*O. ramosa *system [4] and a proteomic study of pea-*O. crenata *interaction [1]. Nevertheless, the exact implication of chitinases during plant-parasitic plant interactions remains unclear.

Thaumatin-like proteins are typical pathogenesis-related proteins with antifungal activity known to be involved in various pathogenic and symbiotic interactions [35,54]. Analysis of their expression pattern in plants responding to different stresses correlates with higher levels of tolerance and resistance [56,57]. We have identified two of these PR proteins: TC94274 (spots 4 and 6) most represented in root tissue of SA 4027, and TC59501 in SA 27774 plants. The fact that all these proteins present increased levels after *O. crenata *infection could indicate that they belong to the general battery of defenses that plants possess, and may eventually take part in defense mechanisms against *Orobanche *upon parasite infection.

#### Glycine-rich and Glycosyl hydrolases proteins

Glycine-rich proteins are involved in cell wall modifications as they have a defensive role as physical barriers, making the host cell more resistant to infection and penetration [38]. Notably, proteins belonging to this group were represented by three different spots: O22385 most represented in SA 27774, TC59317 and Q9SP10, which were modified upon infection in SA 4087 and SA 27774 plants, respectively. Glycine-rich RNA-binding proteins (GR-RBPs) are thought to play an important role in post-transcriptional regulation of gene expression. An increase of protein in pea plants has been reported in response to their infection with *Peronospora viciae*, providing evidence of its role in the response of plants to pathogens [39]. We can speculate that this protein could take an active part in the defense response of the plant by making the host cell more resistant to infection and penetration.

Glycoside hydrolase proteins are involved in the degradation of cell wall polysaccharides. Many studies have revealed changes in cell walls which occur during cell division, expansion, and differentiation and in response to environmental stresses: i.e. pathogens or mechanical stress [58]. This protein (Q2HU16) was modified upon infection in SA 4087 plants.

#### Beta VI allergen and Cysteine protease

Beta VI allergen TC68012 and cysteine protease Q9STA4 were detected at increased levels in healthy SA 4087 root tissues. Members of the Beta VI allergen protein family, which includes intracellular pathogenesis-related proteins, are involved in several defense reactions. They are heavily induced under disease stress and senescence, and some of them have been found to respond to external stimuli related to pathogen defense such as salicylic acid [59]. In addition, some beta allergens from soybean have a remarkable, stable trypsin inhibitor activity, which might be an essential feature for their roles as allergens and to defend themselves from herbivores [60-63]. On the other hand, plant cysteine proteases play a role in a number of processes, such as a nutritional one in reserve development and fruit ripening, degradation of storage proteins in germinating seeds, proenzyme activation, and defective protein degradation. Besides this, they have been implicated in the defense against predators through the degradation of exogenous proteins [64,65].

#### Chalcone-flavone isomerase

The chalcone-flavone isomerase (TC69564) was only detected in roots of non-inoculated SA 4087 genotype. This is the second enzyme of the flavonoid-isoflavonoid pathways in legumes leading to phytoalexin production, whose induction and accumulation is a typical defense response associated with resistance in several plant systems and against a diverse range of attacking organisms [13,66,67]. Biochemical and histological studies in several plant-parasitic plant interactions revealed that production and secretion of these toxic compounds are responsible for parasite development arrest [13,17]. In agreement with these data, several gene expression studies have revealed the induction of the enzymes involved in the phenylpropanoid pathway in response to *Orobanche *infection [68]. In addition, transcriptomic analysis monitoring gene expression patterns of *M. truncatula *root tissue in reponse to *O. crenata *has also identified genes involved in this pathway (Dita MA, Die J, Román B, Krajinski F, Kuster H, Moreno M, Cubero J, Rubiales D: Gene expression profiling of *Medicago truncatula *roots in response to the parasitic plant *Orobanche crenata*, submitted). Even though the identification of chalcone-flavone isomerase in our analysis does not necessarily imply higher production of phytoalexins in SA 4087 plants, it is in harmony with the key role of these secondary metabolites in parasite interaction and resistance.

#### Redox defense-related and signaling

Glutathione S-transferase (GST) TC59483, was found in roots of SA4087 after infection. GSTs are ubiquitous proteins in plants that catalyse the addition of the tripeptide glutathione to a number of electrophilic compounds and constitute one of the primary defense mechanisms against xenobiotics [69]. Members of this family have long been used as stress markers, as their induction precedes the accumulation of PR transcripts upon pathogen infection and several other environmental stresses associated with the generation of ROS [37,49,70,71]. In two recent studies, Jones et al. [72,73] reported the identification by 2-DE electrophoresis of several *Arabidopsis *GSTs showing signs of post-translational modifications in response to *Pseudomonas *infection and in relation to resistance. Members of this family have also been targeted in several non-directed global analyses of plan-parasitic plant interactions using transcriptomic [24] and proteomic approaches [1]. In our analysis, the identification of GST could reflect its role in the control of the redox state of the cell under oxidative stress conditions, due to parasite penetration, and the shift in the p*I *value compared to the theoretical value may be due to the occurrence of some PTMs.

Aldose/aldehyde reductases are also considered to be important factors for scavenging ROS and their toxic products, generated as a consequence of environmental stresses, which can cause cell damage. These enzymes have been described for many organisms, including plants [74]. This is the case of a stress-induced aldehyde reductase from alfalfa, whose ectopic expression in tobacco resulted in tolerance to oxidative stress [75]. Reactive oxygen species (ROS) production and accumulation are involved in the interaction between plant and parasitic plants and resistance [15]. As stated above, it is likely that this aldehyde reductase TC59970 detected at higher levels in roots of healthy SA 4087 plants could play a role in detoxification during parasite infection in a similar way to that reported for other stresses.

#### Cyclophilins

Two different cyclophilins were identified in our study: Q8VX73 more abundant in SA 4087 plants, and MtD20401, detected only in SA 27774. Cyclophilins, members of the peptidyl propyl cis-trans isomerases (PPIase) family, are involved in protein folding by catalysing the cis-trans isomerisation of proline imidic peptide bonds in oligopeptides [76-78]. It is interesting to mention that two recent studies in *Brassica *[79] and *Arabidopsis *[73,80] have reported the involvement of these proteins in incompatible plant-pathogen interactions. Nevertheless, the functional implication of this protein during *Orobanche *infection and resistance is a matter of speculation.

### Proteins belonging to other functional groups

Two spots showing a significant increase in parasite-responding plants correspond to metabolic enzymes. Fructose-bisphosphate aldolase (O65735), increase in intensity in inoculated SA 4087 plants. This enzyme catalyses one of the reactions of the glycolytic/gluconeogenic pathways, i.e. the cleavage of fructose-1,6-bisphosphate to glyceraldehyde-3-phosphate and dihydroxyacetone phosphate. This protein was already found in our previous proteomic analysis of pea-*Orobanche *interaction [1], fructose-bisphosphate aldolase as being most represented in infected susceptible pea plants [1]. In addition, recent studies investigating *Brassica *and pea response to fungal infections at the proteome level report the identification of key enzymes of the photosynthesis, and the resistant genotype's sugar metabolisms were observed to be elevated upon pathogen infection [79,81]. We can speculate that the identification of this enzyme in larger amounts in inoculated SA 4087 plants could imply an increased catabolism in the host root plant to compensate for the cost of resistance [81,82].

Other proteins identified in this work correspond to different functional groups and their relation to plant responses to parasitic plants is unclear. These include: proteasome subunit alpha type 6 (O48551), present in SA 4087 genotype and proteasome subunit alpha type 7 (Q9SXU1), decreasing in inoculated plants of this genotype. These proteins take part in degradation processes of targeted proteins during a number of cell processes, for example in proteolysis and signaling pathways [83,84]. Recently, the proteoasome activity has been correlated with the activation of plant defense reactions [85].

A guanine nucleotide-binding subunit beta-like protein (O24076) was identified in larger amounts in inoculated SA 4087 plants. Recent genetic studies have demonstrated the involvement of G proteins in abscisic acid (ABA) and brassinosteroid sensitivity during seed germination and early plant development, stomatal regulation, D-Glc signaling, light perception, rosette leaf, flower, and silique development, plant defense against necrotrophic fungi and auxin signaling in roots [86].

A general model can be given about proteins found in both genotypes. Defense-and stress-related proteins have been constitutively identified in both genotypes, being many of them predicted to be secreted. However the late resistant genotype (SA 4087) displays a constitutively higher content of proteins of a general response, such as chitinase, cysteine protease, chalcone-flavone isomerase, aldehyde reductase, beta-VI allergen, some of them being previously reported in pea-broomrape interaction [1]. These proteins are part of the typical defense responses induced in response to the attack by a broad range of organisms. In addition, some of these proteins were found to be at higher levels following the parasite infection. This is the case of chitinase and glutathione S-transferase, the latter being related to several environmental stresses associated with the generation of ROS.

Furthermore, the early resistant genotype (SA 27774) seems to have a more targeted response. In addition to some of the proteins mentioned above, several members of the protease inhibitors family were found to be overrepresented, including many trypsin inhibitors, especially in inoculated plants. These results support the implication of these proteins in the early plant response against infection through the secretion of a battery of proteins belonging to this family, which would inhibit the action of proteolytic enzymes secreted by the parasite. However, experiments with soluble secretome and apoplastic proteins are needed to confirm this hypothesis.

## Conclusion

In recent years, proteomic tools have emerged as a powerful complement to transcriptomic approaches to studying the function and regulation of genes and their products. We have accomplished a combination of two-dimensional gel electrophoresis and MALDI-TOF/TOF mass spectrometry analysis to compare the protein changes in roots of two *M. truncatula *accessions displaying different resistant phenotypes, in the hope of contributing to a better understanding of the molecular basis of this plant-parasitic plant interaction and those underlying resistance in legumes. Irrespective of the parasite inoculation, most protein differences were detected between the root protein extracts of the two genotypes, among which several proteins known to be related to biotic and abiotic stresses were identified. Upon *O. crenata *inoculation, alterations in the proteome corresponded to a general increase in the amounts of proteins belonging to the defense-related category, such as proteinase inhibitors, PRs, cell walls modifying, ROS detoxifying enzymes, and enzymes involved in the synthesis of secondary metabolites. The molecular data presented here are in agreement with results obtained for plant-parasitic plant interaction in other systems using proteomics, transcriptomic, biochemical and histological analysis. Moreover, our proteome data correlate with some of the changes detected in the steady-state levels of the transcript from a transcriptomic analysis of exactly the same system and makes sense in the overall context of our system. We have focused on trypsin inhibitor proteins, which, together with the protease inhibitor, have been widely identified in this work, showing majority changes at 21 dpi. Complementary assays have been performed to validate these results. Gel-trypsin inhibition activity confirmed the results previously obtained by mass spectrometry analysis.

The data presented in this work suggest the existence of a generic defense mechanism operating during the early stages of infection, including reinforcement of host cells and the creation of toxic environment for the parasite.

We propose that in SA 27774 a different early mechanism of defense might be operating to the one that takes place in SA 4087. This early defense would depend on proteins targeting key elements for *O. crenata *infection, such as members of the protease inhibitor family, preventing host tissue penetration and connection to the vascular system.

The identification of the elements involved in defense during this interaction could be of crucial importance in helping and directing programs aimed at improving new crop varieties by means of plant breeding and biotechnology. Global monitoring analysis such as this provides an excellent source for candidate elements involved in resistance mechanisms. Nevertheless, it is important to bear in mind that the functional characterization and implication of these proteins in actively responding to parasite infection need to be further validated by means of reverse genetics, using T-DNA or TILLING mutant collections currently established for different legume species.

## Methods

### Plant material, growth conditions and inoculation

Two *Medicago truncatula *genotypes, SA 27774 and SA 4087, kindly provided by the Australian Medicago Genetic Resource Centre (SARDI), were used. They were selected on the basis of differences in behavior against *Orobanche crenata*, SA 27774 being very resistant based on early-acting resistance mechanisms [31], and SA 4087 the most susceptible genotype identified so far [32] but still having a high degree of late-acting resistance [31] (additional file 16).

*O. crenata *seeds were collected from infected pea fields and stored in darkness at room temperature in a desiccator. The viability of *Orobanche *seeds was determined by the 2,3,5-triphenyltetrazolium chloride test [87]. A germination test with the stimulant GR-24, a synthetic analogue of strigolactone, was performed as reported previously [88], the *in vitro *percentage of germination being about 60%.

*M. truncatula *seeds were mechanically scarified and stratified at 8°C for two days to induce germination, and then germinated at 20°C in darkness. Germinated seeds were individually grown in 12 × 12 cm Petri dishes filled with perlite and covered with glass fibre filter paper [89]. The dishes were placed vertically in trays containing Hoagland solution to supply the plants with water and nutrients. The trays were kept in a growth chamber under the following conditions: 20 ± 2°C temperature, 12 h photoperiod (275 μmol m^-2 ^s^-1 ^light intensity) and 80% relative humidity.

Fifteen-day old *M. truncatula *plants were inoculated by spreading 50 mg of *Orobanche *seeds along the roots, resulting in about 115 seeds/cm^2^. *O. crenata *seeds were previously sterilized by immersing them in 0.5% formalin (1% Tween-20) for 140 min. Infection was monitored daily in 10 plants of each genotype under a dissecting microscope at 30× magnification. *O. crenata *seed germination and attachment were observed and quantified 21 days after inoculation, while nodules were observed and counted 4 days later. Four hundred *Orobanche *seeds, close (< 3 mm) to the *M. truncatula *roots, were visualized in each Petri dish, to determine the percentage of germination and contact established with the host roots. Seeds with an emerged radicle were scored as germinated and radicles contacting a host root were recorded as attached.

Root tissues were sampled at 21 and 25 days post-infection based on the microscope observations showing that differences between genotypes were observed at the point between *Orobanche *attachment (21 days post-inoculation) and nodule formation (25 days post-infection). Three plants per sample (genotype, treatment and sampling time) were collected for root protein extractions. Plants were taken from the Petri dish and abundantly washed with water in order to remove all *Orobanche *seeds and non-attached radicles. Nodules, if present, were also removed. Roots were cut at the crown, abundantly washed with water, dried with filter paper, weighed, frozen in liquid nitrogen, and lyophilized prior protein extraction.

### Protein extraction and two-dimensional gel electrophoresis

Proteins from lyophilized root tissues corresponding to 3 plants (0.8–1.8 g root fresh weight) were phenol-extracted and re-suspended according to Dumas-Gaudot et al. [90]. Protein content in the supernatant was quantified by the Bradford method, as reported by Ramagli and Rodriguez [91], using BSA as a standard. Samples were stored at -20°C before electrophoresis.

Precast 18 cm non-linear pH 3–10 (BioRad) were rehydrated for 16 h with 500 μg of proteins (for Coomassie-stained gels), or with 100 μg of proteins (for silver-stained gels) in 350 μl of rehydration buffer containing 8 M urea, 2% (w/v) CHAPS, 20 mM DTT, 2% (v/v) IPG Buffer pH 3–10 and bromophenol blue. Electrofocusing was performed using the Protean IEF Cell system (BioRad) as previously described [1]. After isoelectric focusing, IPG strips were equilibrated according to Görg et al. [92]. Strips were then transferred onto vertical slab 12% SDS-polyacrylamide gels and electrophoresis was run as described by Castillejo et al. [1].

Gels were stained with Coomassie Brilliant Blue G-250 (BioRad) [42] and silver compatible with MS [93]. Gel images were obtained by a SHARP JX-330 scanner and GS800 densitometer (BioRad), and analyzed with the PD-Quest™ software (BioRad), using a 10-fold over background as minimum criteria for spot presence/absence. Normalized spot volumes (individual spot intensity/normalization factor, calculated for each gel based on total quantity in valid spots) were determined for each spot, and these values were used to designate the significant differentially-expressed spots. Only those spots that showed statistically significant differences in intensity as calculated using Student's test (p < 0.05) were considered for further analysis.

### Mass spectrometry analysis and database searching

Protein spots were manually excised from gels. Spots from Coomassie gels were washed with 100 μl of 50% acetonitrile/50 mM ammonium hydrocarbonate pH 8, while spots from silver gels were washed with 50 μl of 15 mM potassium hexacyanoferrate/50 mM sodium thiosulfate [94]. Gel pieces were then dehydrated with acetonitrile and vacuum dried. After rehydratation in 10 μl of 50 mM ammonium hydrocarbonate pH 8, containing 0.5 μg of porcine trypsin (Promega, France), samples were incubated overnight (16–18 h) at 37°C. Peptide fragments from digested proteins were then crystallized with α-cyano-4-hydroxycinnamic acid as a matrix and subjected to MALDI-TOF/TOF-MS (Applied Biosystems, Voyager DE super STR) for peptide mass fingerprinting This instrument is equipped with N_2 _laser (337 nm, Laser of 20 Hz). Samples were acquired in positive reflectron mode with a delay of extraction time of 130 ns. The trypsin autodigestion peaks at 842.509 and 2211.104 were used for internal calibration. The most abundant peptide ions from PMF were then subjected to a new fragmentation (MSMS), providing information that can be used to determine the sequence.

The PMF searches for proteins were performed using EST and protein databases. In the first case, two clustered EST *M. truncatula *databases available online  were used. The first, named MtC, contained 6350 clusters defined from three root EST libraries (24347 ESTs) from a Genoscope project . The clustering process has been previously described by Journet et al. [95]. The second, named MtD, was obtained using the same process on the *M. truncatula *ESTs (approximately 226923 ESTs) available at the Institute for Genomic Research . It contained 21,400 clusters defined from EST libraries corresponding to different *M. truncatula *tissues. A combined search (MS + MSMS) with the PMF (MS) together with data from MSMS fragmentation (up to 3 peptides per protein) was performed in a protein database (SwissProt), using Mascot software  within peptide masses ranging from 700 to 4000 Da (additional file 15). For peptide matching, a minimum of four peptides matches, a maximum of one miscleavage, and peptide modifications by carbamidomethylcysteine and methionine oxidation were accepted. The maximum tolerance for peptide mass matching was limited to 20 ppm. The confidence in the peptide mass fingerprinting matches was based on the score level and confirmed by the accurate overlapping of the matched peptides with the major peaks of the mass spectrum.

N-terminal signal sequences were determined using the Signal P  program.

### In-gel trypsin inhibition assays

Proteins from root tissue (1 g root fresh weight) were extracted with 100 mM Tris-HCl pH7, 5% (w/v) PVPP, and precipitated with methanol/chloroform protocol [96]. Pellets were dissolved in sample buffer [97] to contain 1 μg protein μl^-1^. Analysis in SDS-PAGE slabs containing 0.1% gelatine and 10% acrylamide was performed as described by Heussen and Dowdle [98]. Samples containing 20 μg were heated at 100°C for 3 min and then run on slab gels 1 mm thick. After washing with 25 mM Tris-HCl pH 8.0 containing 1% Triton X-100 and subsequently 10 mM CaCl_2 _to remove SDS, gels were incubated in 40 μg ml^-1 ^trypsin solution for 3 h at 37°C. Then they were washed with distilled water and stained with Coomassie Brilliant Blue. Blue-stained bands revealed a protease inhibitor activity, while a transparent background indicated a complete proteolytic digestion of gelatine. For analysis under non-denaturing conditions, 20 μg of protein were dissolved in sample buffer without reductors and without heating.

## Authors' contributions

MAC carried out the molecular proteomics studies, including 2DE assays, image and statistical analysis, search and acquisition of data, analysis and interpretation of data and drafted the manuscript. AMM drafted the manuscript, EDG participated in the design of the study and participated in the search and acquisition of data, MFA carried out the assays of plant growth in chamber, RS helped to performed the 2DE assays, DR participated in the design of the study and help in the drafting of the manuscript, JJ participated in the design of the study and helped to draft the manuscript. All authors have read and approved the final manuscript.

## Supplementary Material

Additional file 1 ***Orobanche crenata *development on *Medicago truncatula *roots from SA 4087 (A, C, E) and SA 27774 (B, D, F) accessions**. Pictures of germination and attachment were taken at 21 dpi (A, B), and tubercle development 25 dpi (C-F). Pictures D and F show how *Orobanche *failed to establish nodules (pointed out by arrows). Click here for file

Additional file 2 **Coomassie-stained 2-DE gels from root extracts of SA 4087 and SA 27774 plants**. Representative 2-DE gel (up) has been divided into areas A, B, C and D that are enlarged below. Spots showing changes between both genotypes are indicated: circles indicate new or missing spots, triangles and reversed triangles the spots with increased and decreased intensity, respectively. Proteins were resolved on first-dimension, pH 3–10 non-linear gradient, and second dimension, SDS-PAGE on a 12% gel. Molecular mass and p*I *were calculated using the PD-Quest software and standard molecular weight markers. Click here for file

Additional file 3 **Coomassie-stained 2-DE gels from root extracts of control, non-inoculated and inoculated SA 4087 plants**. Representative 2-DE gel (up) has been divided into areas A, B, C and D that are enlarged below. Spots showing changes between treatments are indicated: circles indicate new or missing spots, triangles and reversed triangles the spots with increased and decreased intensity, respectively. Proteins were resolved on first-dimension, pH 3–10 non-linear gradient, and second dimension, SDS-PAGE on a 12% gel. Molecular mass and p*I *were calculated using the PD-Quest software and standard molecular weight markers. Click here for file

Additional file 4 **Coomassie-stained 2-DE gels from root extracts of control, non-inoculated and inoculated SA 27774 plants**. Representative 2-DE gel (up) has been divided into areas A, B, C and D that are enlarged below. Spots showing changes between treatments are indicated: circles indicate new or missing spots, triangles and reversed triangles the spots with increased and decreased intensity, respectively. Proteins were resolved on first-dimension, pH 3–10 non-linear gradient, and second dimension, SDS-PAGE on a 12% gel. Molecular mass and p*I *were calculated using the PD-Quest software and standard molecular weight markers. Click here for file

Additional file 5 **Silver-stained 2-DE gels from root extracts of SA 4087 and SA 27774 plants**. Representative 2-DE gel (up) has been divided into areas A, B, C and D that are enlarged below. Spots showing changes between both genotypes are indicated: circles indicate new or missing spots, triangles and reversed triangles the spots with increased and decreased intensity, respectively. Proteins were resolved on first-dimension, pH 3–10 non-linear gradient, and second dimension, SDS-PAGE on a 12% gel. Molecular mass and p*I *were calculated using the PD-Quest software and standard molecular weight markers. Click here for file

Additional file 6 **Silver-stained 2-DE gels from root extracts of control, non-inoculated and inoculated SA 4087 plants**. Representative 2-DE gel (up) has been divided into areas A, B, C and D that are enlarged below. Spots showing changes between treatments are indicated: circles indicate new or missing spots, triangles and reversed triangles the spots with increased and decreased intensity, respectively. Proteins were resolved on first-dimension, pH 3–10 non-linear gradient, and second dimension, SDS-PAGE on a 12% gel. Molecular mass and p*I *were calculated using the PD-Quest software and standard molecular weight markers. Click here for file

Additional file 7 **Silver-stained 2-DE gels from root extracts of control, non-inoculated and inoculated SA 27774 plants**. Representative 2-DE gel (up) has been divided into areas A, B, C and D that are enlarged below. Spots showing changes between treatments are indicated: circles indicate new or missing spots, triangles and reversed triangles the spots with increased and decreased intensity, respectively. Proteins were resolved on first-dimension, pH 3–10 non-linear gradient, and second dimension, SDS-PAGE on a 12% gel. Molecular mass and p*I *were calculated using the PD-Quest software and standard molecular weight markers. Click here for file

Additional file 8 **Quantitative data for the spots detected in Coomassie stained gels showing differences between genotypes**. Click here for file

Additional file 9 **Quantitative data for the spots detected in Coomassie stained gels showing differences between control and inoculated SA 4087 plants**. Click here for file

Additional file 10 **Quantitative data for the spots detected in Coomassie stained gels showing differences between control and inoculated SA 27774 plants**. Click here for file

Additional file 11 **Quantitative data for the spots detected in silver stained gels showing differences between genotypes**. Click here for file

Additional file 12 **Quantitative data for the spots detected in silver stained gels showing differences between control and inoculated SA 4087 plants**. Click here for file

Additional file 13 **Quantitative data for the spots detected in silver stained gels showing differences between control and inoculated SA 27774 plants**. Click here for file

Additional file 14 **Analysis of variance of spots differentially expressed**. Click here for file

Additional file 15 **List of identified proteins**. Click here for file

Additional file 16 **Root growth of *Medicago truncatula *SA 4087 and SA 27774 control non-inoculated and inoculated plants**. Click here for file
