# Supplementary figures and images for: Differential expression proteomics to investigate responses and resistance to Orobanche crenata in Medicago truncatula
Source: BMC Genomics. 2009 Jul 3;10:294. doi: 10.1186/1471-2164-10-294 (PMC2714000; doi:10.1186/1471-2164-10-294)

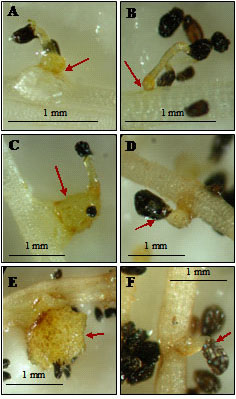

Supplement: Additional file 1 — Orobanche crenata development on Medicago truncatula roots from SA 4087 (A, C, E) and SA 27774 (B, D, F) accessions. Pictures of germination and attachment were taken at 21 dpi (A, B), and tubercle development 25 dpi (C-F). Pictures D and F show how Orobanche failed to establish nodules (pointed out by arrows). [file 1471-2164-10-294-S1.jpeg]

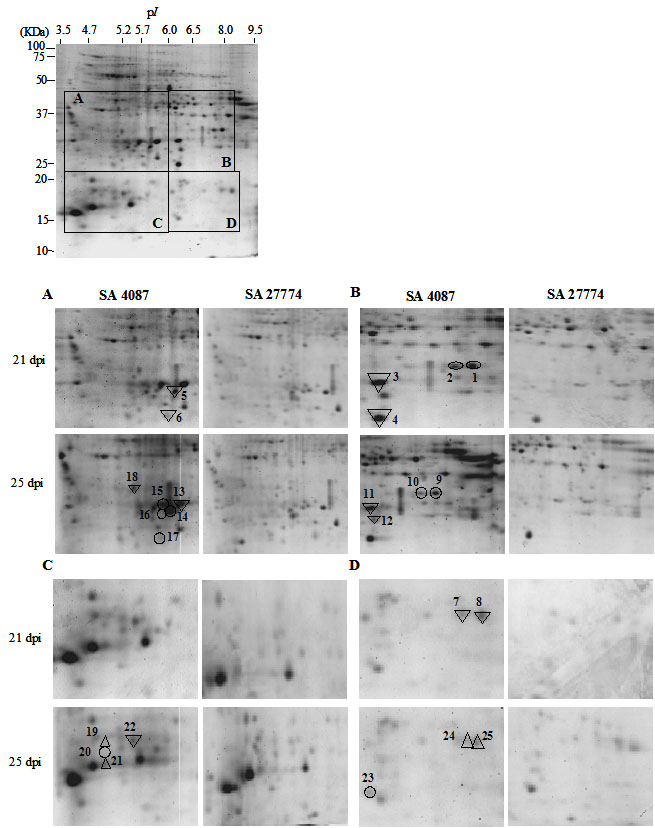

Supplement: Additional file 2 — Coomassie-stained 2-DE gels from root extracts of SA 4087 and SA 27774 plants. Representative 2-DE gel (up) has been divided into areas A, B, C and D that are enlarged below. Spots showing changes between both genotypes are indicated: circles indicate new or missing spots, triangles and reversed triangles the spots with increased and decreased intensity, respectively. Proteins were resolved on first-dimension, pH 3–10 non-linear gradient, and second dimension, SDS-PAGE on a 12% gel. Molecular mass and pI were calculated using the PD-Quest software and standard molecular weight markers. [file 1471-2164-10-294-S2.jpeg]

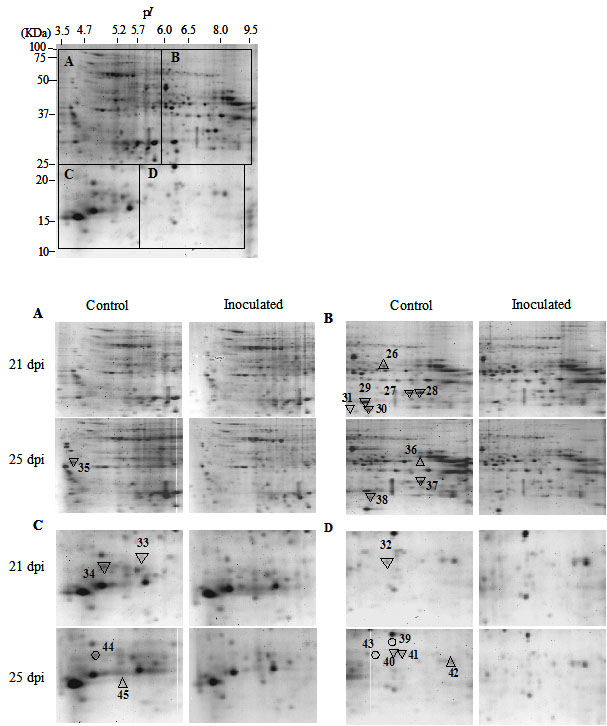

Supplement: Additional file 3 — Coomassie-stained 2-DE gels from root extracts of control, non-inoculated and inoculated SA 4087 plants. Representative 2-DE gel (up) has been divided into areas A, B, C and D that are enlarged below. Spots showing changes between treatments are indicated: circles indicate new or missing spots, triangles and reversed triangles the spots with increased and decreased intensity, respectively. Proteins were resolved on first-dimension, pH 3–10 non-linear gradient, and second dimension, SDS-PAGE on a 12% gel. Molecular mass and pI were calculated using the PD-Quest software and standard molecular weight markers. [file 1471-2164-10-294-S3.jpeg]

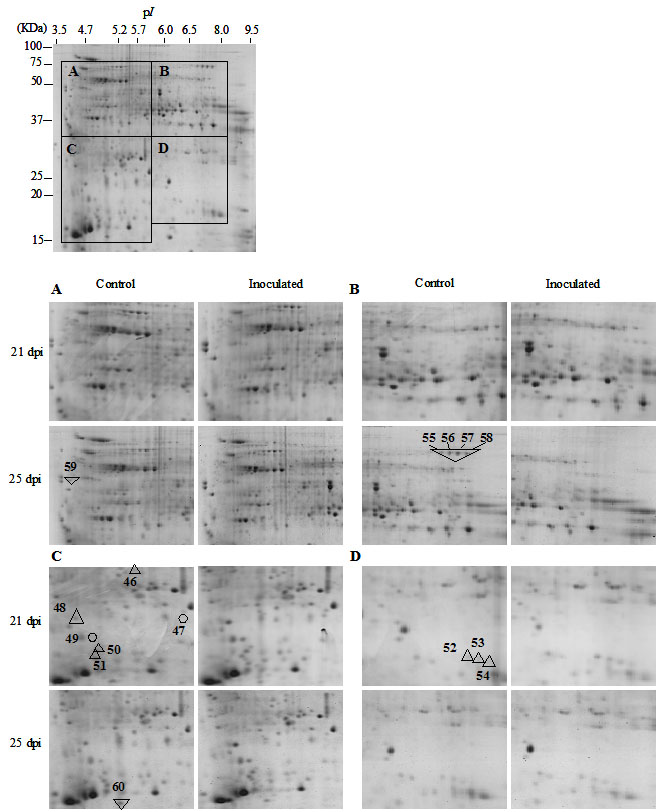

Supplement: Additional file 4 — Coomassie-stained 2-DE gels from root extracts of control, non-inoculated and inoculated SA 27774 plants. Representative 2-DE gel (up) has been divided into areas A, B, C and D that are enlarged below. Spots showing changes between treatments are indicated: circles indicate new or missing spots, triangles and reversed triangles the spots with increased and decreased intensity, respectively. Proteins were resolved on first-dimension, pH 3–10 non-linear gradient, and second dimension, SDS-PAGE on a 12% gel. Molecular mass and pI were calculated using the PD-Quest software and standard molecular weight markers. [file 1471-2164-10-294-S4.jpeg]

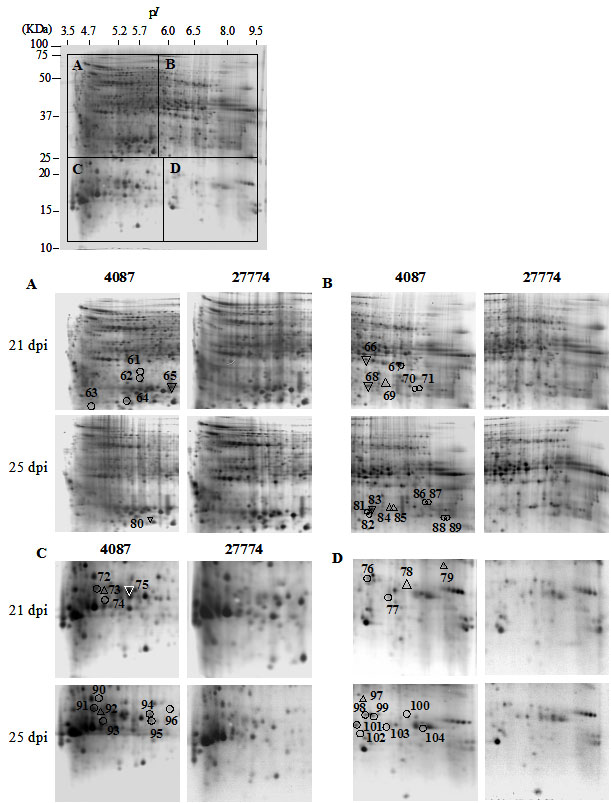

Supplement: Additional file 5 — Silver-stained 2-DE gels from root extracts of SA 4087 and SA 27774 plants. Representative 2-DE gel (up) has been divided into areas A, B, C and D that are enlarged below. Spots showing changes between both genotypes are indicated: circles indicate new or missing spots, triangles and reversed triangles the spots with increased and decreased intensity, respectively. Proteins were resolved on first-dimension, pH 3–10 non-linear gradient, and second dimension, SDS-PAGE on a 12% gel. Molecular mass and pI were calculated using the PD-Quest software and standard molecular weight markers. [file 1471-2164-10-294-S5.jpeg]

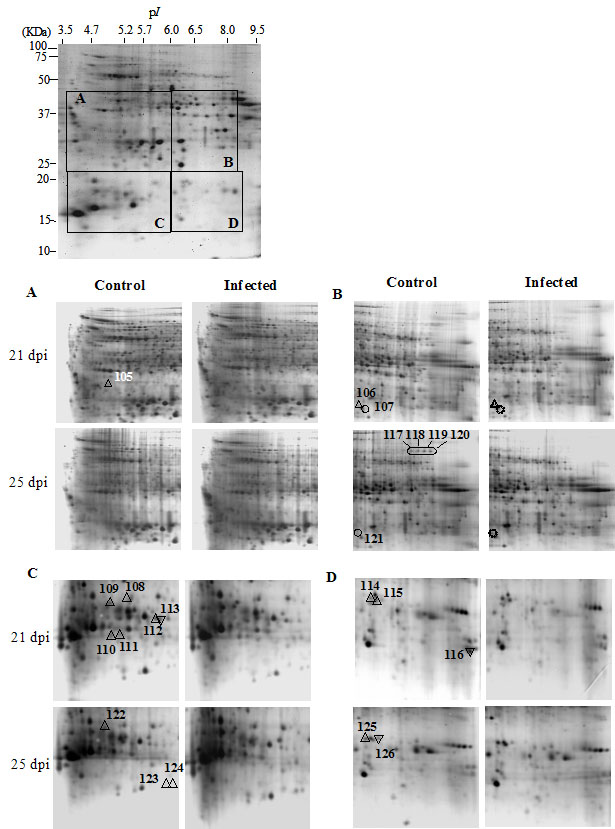

Supplement: Additional file 6 — Silver-stained 2-DE gels from root extracts of control, non-inoculated and inoculated SA 4087 plants. Representative 2-DE gel (up) has been divided into areas A, B, C and D that are enlarged below. Spots showing changes between treatments are indicated: circles indicate new or missing spots, triangles and reversed triangles the spots with increased and decreased intensity, respectively. Proteins were resolved on first-dimension, pH 3–10 non-linear gradient, and second dimension, SDS-PAGE on a 12% gel. Molecular mass and pI were calculated using the PD-Quest software and standard molecular weight markers. [file 1471-2164-10-294-S6.jpeg]

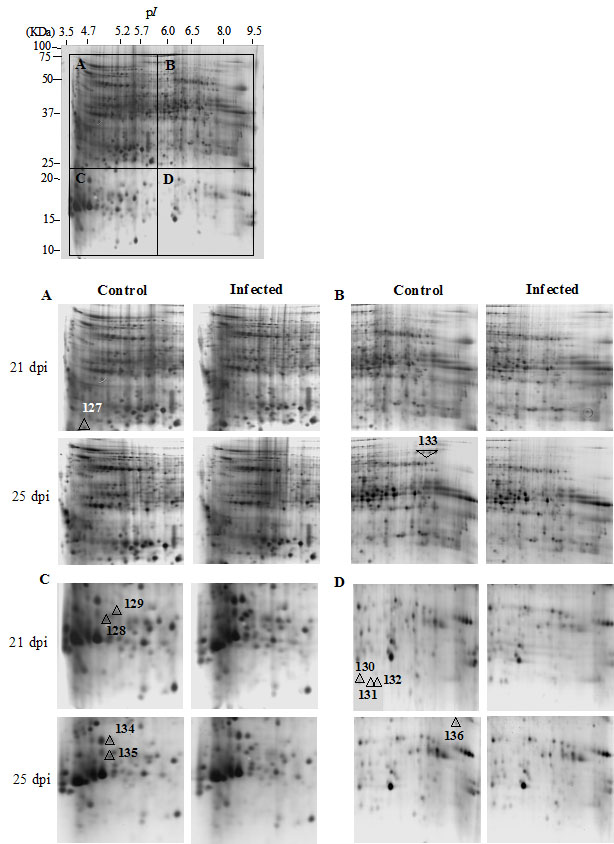

Supplement: Additional file 7 — Silver-stained 2-DE gels from root extracts of control, non-inoculated and inoculated SA 27774 plants. Representative 2-DE gel (up) has been divided into areas A, B, C and D that are enlarged below. Spots showing changes between treatments are indicated: circles indicate new or missing spots, triangles and reversed triangles the spots with increased and decreased intensity, respectively. Proteins were resolved on first-dimension, pH 3–10 non-linear gradient, and second dimension, SDS-PAGE on a 12% gel. Molecular mass and pI were calculated using the PD-Quest software and standard molecular weight markers. [file 1471-2164-10-294-S7.jpeg]
