# Supplementary material for: Differential expression proteomics to investigate responses and resistance to Orobanche crenata in Medicago truncatula
Source: BMC Genomics. 2009 Jul 3;10:294. doi: 10.1186/1471-2164-10-294 (PMC2714000; doi:10.1186/1471-2164-10-294)
Supplement: Additional file 8 — Quantitative data for the spots detected in Coomassie stained gels showing differences between genotypes. [file 1471-2164-10-294-S8.doc]

Differential protein spots between Coomassie stained 2-DE gels from roots of control, non-inoculated, SA4087 and SA27774 plants

| **Spot number** | **Gel area^a^** | **Experimental^b^**  ***Mr* (kDa) p*I*** | | **Normalized Volume^bc^ x ± SD**  **SA4087 SA27774** | | | |
| --- | --- | --- | --- | --- | --- | --- | --- |
| 1* | B | 33.3 | 7.5 | 6767.7 ± | 5106 | nd^d^ |  |
| 2* | B | 33.3 | 6.9 | 7119.8 ± | 1485 | nd^d^ |  |
| 3* | B | 31.0 | 6.1 | 13255.3 ± | 3564 | 943.4 ± | 588 |
| 4* | B | 23.4 | 6.1 | 25669.0 ± | 2761 | 11151.7 ± | 1891 |
| 5 | A | 28.2 | 5.7 | 10614.8 ± | 2118 | 3145.2 ± | 693 |
| 6* | A | 22.5 | 5.6 | 5912.7 ± | 674 | 3108.9 ± | 482 |
| 7* | D | 18.5 | 7.4 | 4315.2 ± | 139 | 1046.9 ± | 446 |
| 8* | D | 18.5 | 8.0 | 8023.7 ± | 1572 | 1124.1 ± | 157 |
| 9 | B | 31.9 | 7.4 | 9316.9 ± | 1201 | 1307.7 ± | 418 |
| 10 | B | 31.8 | 6.8 | 7563.3 ± | 2522 | 1013.3 ± | 84 |
| 11 | B | 29.3 | 6.1 | 13863.4 ± | 7005 | 1067.8 ± | 54 |
| 12 | B | 26.8 | 6.1 | 6438.6 ± | 1757 | 918.5 ± | 55 |
| 13 | A | 28.8 | 5.8 | 16253.5 ± | 4980 | 523.1 ± | 60 |
| 14 | A | 27.3 | 5.7 | 13093.5 ± | 501 | nd^d^ |  |
| 15* | A | 28.7 | 5.6 | 6044.9 ± | 841 | nd^d^ |  |
| 16* | A | 29.6 | 5.4 | nd^d^ |  | 9378.5 ± | 1486 |
| 17 | A | 25.7 | 5.3 | nd^d^ |  | 6674.2 ± | 509 |
| 18* | A | 31.1 | 5.3 | 6367.0 ± | 1468 | 1389.4 ± | 358 |
| 19* | C | 17.7 | 5.0 | 539.4 ± | 237 | 2726.7 ± | 640 |
| 20 | C | 17.1 | 4.8 | nd^d^ |  | 3123.5 ± | 964 |
| 21* | C | 16.7 | 5.0 | 1540.9 ± | 851 | 6435.3 ± | 912 |
| 22* | C | 17.9 | 5.3 | 7801.1 ± | 1769 | 1370.5 ± | 163 |
| 23* | D | 15.8 | 6.0 | 4036.1 ± | 494 | nd^d^ |  |
| 24 | D | 18.3 | 7.5 | 1074.6 ± | 555 | 3188.3 ± | 1198 |
| 25 | D | 18.2 | 8.1 | 4459.8 ± | 1056 | 8693.1 ± | 2190 |

Only those changes consistently manifested in all the three independent replicates and significantly variable between treatments (P < 0.05) were included.

* indicate identified spots (additional file 15)

a) Localization of spots according to the gel areas defined in figure from additional file 2.

b) Molecular masses (*Mr*) and isoelectric points (p*I*), as well as normalized volumes were calculated with the PD-Quest Software.

c) Values are mean of the three independent replicates.

d) Non-detected
