# Supplementary material for: Differential expression proteomics to investigate responses and resistance to Orobanche crenata in Medicago truncatula
Source: BMC Genomics. 2009 Jul 3;10:294. doi: 10.1186/1471-2164-10-294 (PMC2714000; doi:10.1186/1471-2164-10-294)
Supplement: Additional file 9 — Quantitative data for the spots detected in Coomassie stained gels showing differences between control and inoculated SA 4087 plants. [file 1471-2164-10-294-S9.doc]

Differential protein spots between Coomassie stained 2-DE gels from roots of SA4087 accession in response to the *O. crenata* inoculation

| **Spot number** | **Gel area^a^** | **Experimental^b^**  ***Mr* (kDa) p*I*** | | **Normalized Volume^bc^ x ± SD**  **Control Inoculated** | | | |
| --- | --- | --- | --- | --- | --- | --- | --- |
| 26* | B | 38.5 | 6.3 | 1711.9 ± | 503 | 3384.1 ± | 746 |
| 27* | B | 33.3 | 6.9 | 7119.8 ± | 1485 | 3104.4 ± | 670 |
| 28* | B | 33.3 | 7.5 | 8544.1 ± | 2335 | 2410.5 ± | 1847 |
| 29* | B | 31.0 | 6.1 | 18275.6 ± | 5157 | 6874.4 ± | 2120 |
| 30 | B | 27.8 | 6.1 | 5710.8 ± | 2630 | 1530.8 ± | 605 |
| 31 | B | 27.9 | 5.9 | 930.7 ± | 192 | 347.4 ± | 87 |
| 32 | D | 17.9 | 6.0 | 2883.0 ± | 589 | 979.9 ± | 313 |
| 33* | C | 18.8 | 5.4 | 1824.8 ± | 77 | 584.5 ± | 242 |
| 34 | C | 17.9 | 4.6 | 4671.7 ± | 987 | 1380.3 ± | 284 |
| 35* | A | 38.5 | 4.0 | 4328.7 ± | 1075 | 1508.1 ± | 275 |
| 36* | B | 38.5 | 7.4 | 789.8 ± | 195 | 1653.3 ± | 179 |
| 37 | B | 31.8 | 7.4 | 9316.9 ± | 1201 | 3204.3 ± | 638 |
| 38* | B | 26.8 | 6.1 | 6438.6 ± | 1757 | 1785.1 ± | 843 |
| 39* | D | 21.9 | 6.1 | nd^d^ |  | 2043.0 ± | 798 |
| 40* | D | 19.2 | 6.1 | 1925.7 ± | 384 | 592.1 ± | 210 |
| 41* | D | 19.0 | 6.3 | 4263.5 ± | 2143 | 908.7 ± | 395 |
| 42 | D | 18.2 | 8.1 | 4459.8 ± | 1056 | 12512.1 ± | 2603 |
| 43 | D | 18.8 | 5.9 | nd^d^ |  | 686.8 ± | 69 |
| 44* | C | 18.0 | 4.7 | 4861.4 ± | 3286 | 249.9 ± | 86 |
| 45 | C | 15.7 | 5.3 | 1000.8 ± | 504 | 4711.5 ± | 1112 |

Only those changes consistently manifested in all the three independent replicates and significantly variable between treatments (P < 0.05) were included.

* indicate identified spots (additional file 15)

a) Localization of spots according to the gel areas defined in figure from additional file 3.

b) Molecular masses (*Mr*) and isoelectric points (p*I*), as well as normalized volumes were calculated with the PD-Quest Software.

c) Values are mean of the three independent replicates.

d) Non-detected
