# Supplementary material for: Differential expression proteomics to investigate responses and resistance to Orobanche crenata in Medicago truncatula
Source: BMC Genomics. 2009 Jul 3;10:294. doi: 10.1186/1471-2164-10-294 (PMC2714000; doi:10.1186/1471-2164-10-294)
Supplement: Additional file 10 — Quantitative data for the spots detected in Coomassie stained gels showing differences between control and inoculated SA 27774 plants. [file 1471-2164-10-294-S10.doc]

Differential protein spots between Coomassie stained 2-DE gels from roots of SA27774 accession in response to the *O. crenata* inoculation

| **Spot number** | **Gel area^a^** | **Experimental^b^**  ***Mr* (kDa) p*I*** | | **Normalized Volume^bc^ x ± SD**  **Control Inoculated** | | | |
| --- | --- | --- | --- | --- | --- | --- | --- |
| 46 | C | 34.3 | 5.2 | 480.7 ± | 149 | 1370.2 ± | 612 |
| 47 | C | 26.6 | 5.6 | nd^d^ |  | 1299.2 ± | 790 |
| 48 | C | 26.4 | 4.6 | 1426.3 ± | 147 | 10558.1 ± | 4082 |
| 49 | C | 23.7 | 4.9 | nd^d^ |  | 8164.4 ± | 5008 |
| 50* | C | 22.2 | 4.9 | 1210.6 ± | 499 | 4544.9 ± | 1114 |
| 51* | C | 21.7 | 4.9 | 726.2 ± | 325 | 3091.3 ± | 1407 |
| 52* | D | 23.2 | 6.5 | 1326.4 ± | 396 | 3486.3 ± | 812 |
| 53* | D | 23.0 | 7.1 | 633.0 ± | 218 | 2034.6 ± | 902 |
| 54* | D | 22.6 | 7.6 | 377.7 ± | 162 | 1208.7 ± | 253 |
| 55* | B | 66.9 | 6.6 | 1404.7 ± | 401 | nd^d^ |  |
| 56 | B | 66.6 | 6.9 | 1967.1 ± | 885 | nd^d^ |  |
| 57* | B | 66.4 | 7.2 | 1967.7 ± | 1188 | nd^d^ |  |
| 58* | B | 66.2 | 7.5 | 1516.8 ± | 1111 | nd^d^ |  |
| 59 | A | 50.6 | 3.7 | 909.6 ± | 102 | 542.2 ± | 121 |
| 60* | C | 15.5 | 4.9 | 4844.2 ± | 651 | 2941.1 ± | 700 |

Only those changes consistently manifested in all the three independent replicates and significantly variable between treatments (P < 0.05) were included.

* indicate identified spots (additional file 15)

a) Localization of spots according to the gel areas defined in figure from additional file 4.

b) Molecular masses (*Mr*) and isoelectric points (p*I*), as well as normalized volumes were calculated with the PD-Quest Software.

c) Values are mean of the three independent replicates.

d) Non-detected
