# Supplementary material for: Differential expression proteomics to investigate responses and resistance to Orobanche crenata in Medicago truncatula
Source: BMC Genomics. 2009 Jul 3;10:294. doi: 10.1186/1471-2164-10-294 (PMC2714000; doi:10.1186/1471-2164-10-294)
Supplement: Additional file 11 — Quantitative data for the spots detected in silver stained gels showing differences between genotypes. [file 1471-2164-10-294-S11.doc]

Differential protein spots between silver stained 2-DE gels from roots of control, non-inoculated, SA4087 and SA27774 plants

| **Spot number** | **Gel area^a^** | **Experimental^b^**  ***Mr* (kDa) p*I*** | | **Normalized Volume^bc^ x ± SD**  **SA4087 SA27774** | |
| --- | --- | --- | --- | --- | --- |
| 61 | A | 31.9 | 5.2 | nd^d^ | 1016 ± 181 |
| 62 | A | 30.6 | 5.2 | nd^d^ | 890 ± 78 |
| 63* | A | 22.6 | 4.6 | nd^d^ | 2218 ± 224 |
| 64* | A | 25.0 | 5.0 | 1909 ± 241 | nd^d^ |
| 65 | A | 28.7 | 5.6 | 4430 ± 3010 | 1649 ± 314 |
| 66* | B | 36.7 | 6.0 | 2635 ± 686 | 1137 ± 135 |
| 67 | B | 35.5 | 6.4 | 2044 ± 84 | nd^d^ |
| 68 | B | 28.5 | 6.0 | 5117 ± 613 | 1169 ± 665 |
| 69* | B | 28.6 | 6.3 | 606 ± 82 | 1851 ± 226 |
| 70 | B | 28.6 | 6.7 | nd^d^ | 1254 ± 256 |
| 71 | B | 28.7 | 6.9 | nd^d^ | 1489 ± 680 |
| 72 | C | 18.1 | 4.7 | 1873 ± 914 | nd^d^ |
| 73 | C | 17.6 | 4.8 | 159 ± 12 | 1029 ± 175 |
| 74 | C | 16.9 | 4.8 | nd^d^ | 1478 ± 467 |
| 75* | C | 17.6 | 5.1 | 10486 ± 3172 | 3521 ± 830 |
| 76 | D | 18.9 | 6.1 | 801 ± 280 | nd^d^ |
| 77 | D | 17.6 | 6.3 | nd^d^ | 687 ± 484 |
| 78 | D | 19.0 | 6.5 | 97 ± 18 | 1256 ± 318 |
| 79 | D | 21.5 | 8.4 | 501 ± 178 | 949 ± 125 |
| 80 | A | 25.2 | 5.4 | 829 ± 250 | 229 ± 48 |
| 81 | B | 27.2 | 5.9 | nd^d^ | 1171 ± 421 |
| 82 | B | 26.8 | 5.9 | nd^d^ | 637 ± 171 |
| 83 | B | 27.6 | 6.0 | 4197 ± 1260 | 648 ± 495 |
| 84* | B | 27.6 | 6.3 | 436 ± 5 | 1226 ± 440 |
| 85 | B | 27.5 | 6.4 | 332 ± 73 | 2116 ± 423 |
| 86 | B | 27.8 | 7.1 | nd^d^ | 769 ± 270 |
| 87 | B | 27.9 | 7.2 | nd^d^ | 1639 ± 474 |
| 88 | B | 25.9 | 8.2 | nd^d^ | 708 ± 372 |
| 89 | B | 25.9 | 8.3 | nd^d^ | 429 ± 143 |
| 90 | C | 19.4 | 4.8 | nd^d^ | 1342 ± 70 |
| 91 | C | 18.2 | 4.8 | 2762 ± 682 | nd^d^ |
| 92 | C | 17.9 | 4.9 | 201 ± 60 | 1145 ± 348 |
| 93 | C | 16.9 | 4.9 | nd^d^ | 2697 ± 1738 |
| 94 | C | 17.6 | 5.5 | 1973 ± 155 | nd^d^ |
| 95 | C | 16.9 | 5.5 | 918 ± 328 | nd^d^ |
| 96 | C | 18.2 | 5.8 | 476 ± 196 | nd^d^ |
| 97* | D | 21.6 | 6.0 | 280 ± 21 | 713 ± 235 |
| 98 | D | 18.8 | 6.0 | 1076 ± 75 | nd^d^ |
| 99 | D | 18.7 | 6.2 | 1072 ± 77 | nd^d^ |
| 100 | D | 18.8 | 6.9 | nd^d^ | 735 ± 178 |
| 101 | D | 17.6 | 5.9 | 1881 ± 194 | nd^d^ |
| 102 | D | 16.5 | 5.9 | nd^d^ | 628 ± 240 |
| 103 | D | 17.5 | 6.4 | nd^d^ | 811 ± 325 |
| 104* | D | 17.3 | 7.6 | nd^d^ | 1080 ± 184 |

Only those changes consistently manifested in all the three independent replicates and significantly variable between accessions (P < 0.05) were included

* indicate identified spots (additional file 15)

a) Localization of spots according to the gel areas defined in figure from additional file 5.

b) Molecular masses (*Mr*) and isoelectric points (p*I*), as well as normalized volumes were calculated with the PD-Quest Software.

c) Values are mean of the three independent replicates.

d) Non-detected
