# Supplementary material for: Differential expression proteomics to investigate responses and resistance to Orobanche crenata in Medicago truncatula
Source: BMC Genomics. 2009 Jul 3;10:294. doi: 10.1186/1471-2164-10-294 (PMC2714000; doi:10.1186/1471-2164-10-294)
Supplement: Additional file 12 — Quantitative data for the spots detected in silver stained gels showing differences between control and inoculated SA 4087 plants. [file 1471-2164-10-294-S12.doc]

Differential protein spots between silver stained 2-DE gels from roots of SA4087 accession in response to the *O. crenata* inoculation

| **Spot number^a^** | **Gel area^a^** | **Experimental^b^**  ***Mr* (kDa) p*I*** | | **Normalized Volume^bc^ x ± SD**  **Control Inoculated** | |
| --- | --- | --- | --- | --- | --- |
| 105 | A | 32.9 | 4.8 | 278 ± 139 | 965 ± 240 |
| 106 | B | 26.7 | 5.8 | 163 ± 40 | 627 ± 240 |
| 107* | B | 25.5 | 5.9 | nd^d^ | 450 ± 180 |
| 108 | C | 19.2 | 5.2 | 149 ± 85 | 1288 ± 599 |
| 109 | C | 18.8 | 4.9 | 741 ± 259 | 2658 ± 629 |
| 110* | C | 15.9 | 4.9 | 1080 ± 122 | 2954 ± 325 |
| 111 | C | 16.0 | 5.1 | 298 ± 141 | 1716 ± 937 |
| 112 | C | 17.4 | 5.6 | 754 ± 156 | 2528 ± 447 |
| 113 | C | 17.3 | 5.6 | 2018 ± 791 | 604 ± 43 |
| 114* | D | 19.3 | 6.0 | 660 ± 272 | 2341 ± 567 |
| 115 | D | 19.0 | 6.1 | 862 ± 98 | 2358 ± 482 |
| 116 | D | 14.4 | 9.2 | 4220 ± 784 | 1258 ± 302 |
| 117 | B | 66.4 | 6.7 | 1732 ± 624 | nd^d^ |
| 118 | B | 66.4 | 7.0 | 1413 ± 482 | nd^d^ |
| 119 | B | 66.2 | 7.3 | 1581 ± 602 | nd^d^ |
| 120 | B | 66.1 | 7.5 | 1161 ± 343 | nd^d^ |
| 121 | B | 26.1 | 5.8 | nd^d^ | 644 ± 69 |
| 122 | C | 18.4 | 4.9 | 783 ± 85 | 1963 ± 264 |
| 123 | C | 12.7 | 5.8 | 627 ± 112 | 1322 ± 360 |
| 124 | C | 12.8 | 5.8 | 421 ± 110 | 909 ± 163 |
| 125 | D | 18.8 | 6.0 | 1251 ± 156 | 2277 ± 334 |
| 126 | D | 18.8 | 6.3 | 968 ± 188 | 336 ± 143 |

Only those changes consistently manifested in all the three independent replicates and significantly variable between treatments (P < 0.05) were included.

* indicate identified spots (additional file 15)

a) Localization of spots according to the gel areas defined in figure from additional file 6.

b) Molecular masses (*Mr*) and isoelectric points (p*I*), as well as normalized volumes were calculated with the PD-Quest Software.

c) Values are mean of the three independent replicates.

d) Non-detected
