# Supplementary material for: Differential expression proteomics to investigate responses and resistance to Orobanche crenata in Medicago truncatula
Source: BMC Genomics. 2009 Jul 3;10:294. doi: 10.1186/1471-2164-10-294 (PMC2714000; doi:10.1186/1471-2164-10-294)
Supplement: Additional file 13 — Quantitative data for the spots detected in silver stained gels showing differences between control and inoculated SA 27774 plants. [file 1471-2164-10-294-S13.doc]

Differential protein spots between silver stained 2-DE gels from roots of SA27774 accession in response to the *O. crenata* inoculation

| **Spot number** | **Gel area^a^** | **Experimental^b^**  ***Mr* (kDa) p*I*** | | **Normalized Volume^bc^ x ± SD**  **Control Inoculated** | |
| --- | --- | --- | --- | --- | --- |
| 127* | A | 21.2 | 4.5 | 1858 ± 121 | 6302 ± 2083 |
| 128 | C | 17.2 | 4.7 | 794 ± 199 | 2131 ± 688 |
| 129* | C | 17.8 | 4.8 | 1062 ± 247 | 2129 ± 644 |
| 130 | D | 12.2 | 5.5 | 585 ± 66 | 1165 ± 152 |
| 131 | D | 11.8 | 5.8 | 172 ± 163 | 616 ± 55 |
| 132 | D | 11.9 | 5.9 | 416 ± 84 | 772 ± 61 |
| 133 | B | 68.4 | 6.6 | 1123 ± 384 | 110 ± 176 |
| 134* | C | 18.8 | 4.8 | 1574 ± 111 | 5117 ± 408 |
| 135 | C | 17.5 | 4.8 | 1352 ± 537 | 2911 ± 850 |
| 136 | D | 22.3 | 8.4 | 344 ± 88 | 616 ± 77 |

Only those changes consistently manifested in all the three independent replicates and significantly variable between treatments (P < 0.05) were included.

* indicate identified spots (additional file 15)

a) Localization of spots according to the gel areas defined in figure from additional file 7.

b) Molecular masses (*Mr*) and isoelectric points (p*I*), as well as normalized volumes were calculated with the PD-Quest Software.

c) Values are mean of the three independent replicates.
