# Supplementary material for: Differential expression proteomics to investigate responses and resistance to Orobanche crenata in Medicago truncatula
Source: BMC Genomics. 2009 Jul 3;10:294. doi: 10.1186/1471-2164-10-294 (PMC2714000; doi:10.1186/1471-2164-10-294)
Supplement: Additional file 15 — List of identified proteins. [file 1471-2164-10-294-S15.doc]

List of identified proteins

| **Spot^a^** | | **Change (dpi)^b^** | | **Homologous protein**  **(species)^c^** | **Database entry^c^** | | | | | | | **Stain^d^** | **Matched/ Total peptides^e^** | **Score** | **Peptides sequence^f^** | | | | **Ion Score** | **Sequence**  **coverage (%)^g^** | ***Mr*/p*I*^h^ experimental (theoretical)** | |
| --- | --- | --- | --- | --- | --- | --- | --- | --- | --- | --- | --- | --- | --- | --- | --- | --- | --- | --- | --- | --- | --- | --- |
| **SA27774/SA4087 (E/L)** | | | | | | | | | | | | | | | | | | | | | | |
| **Defense and stress related** | | | | | | | | | | | | | | | | | | | | | | |
| 1* | L (21) | | Chitinase | | | | | | | TC106842 | | C | 11/65 | 296 | WSPSSADR  LYTYDAFISAAK  AFPNFANNGDTATK | | | | 50  80  83 | 36 | | 33.3/7.5 (35.9/6.8) |
| 2* | L (21) | | Chitinase | | | | | | | TC106842 | | C | 13/65 | 272 | WSPSSADR  DTFNNMLK  LYTYDAFISAAK | | | | 50  40  75 | 44 | | 33.3/6.9 (35.9/6.8) |
| 4* | ↓ E (21) | | Thaumatin-like protein PR-5b precursor  (*Cicer arietinum*) | | | | | | | TC94274 | | C | 5/65 | 287 | TGCNFDGSGR  TGCNFDGSGRGR  ISCTADINGQCPNELR | | | | 86  66  103 | 28 | | 23.4/6.1 (26.9/5.3) |
| 6* | ↓ E (21) | | Thaumatin-like protein PR-5b precursor  (*Cicer arietinum*) | | | | | | | TC94274 | | C | 4/65 | 276 | TGCNFDGSGR  TGCNFDGSGRGR  ISCTADINGQCPNELR | | | | 83  77  101 | 11 | | 22.5/5.6 (26.9/5.3) |
| 16 | E (25) | | Disease resistance  (*Oryza sativa*) | | | | | | | Q8H816^◘^ | | C | 10/66 | 76 |  | | | |  | 42 | | 29.6/5.4  (30.8/6.1) |
| 18 | ↓ E (25) | | Cysteine protease  (*Medicago sativa*) | | | | | | | Q9STA4^◘^ | | C | 6/64 | 147 | DYAYTGR  DGSCKFDK  GRNVCGVDSMVSTVAAAQS | | | | 34  57  12 | 26 | | 31.1/5.3 (22.9/5.4) |
| 19 | ↑ E (25) | | Glycine-rich protein  (*Oryza sativa*) | | | | | | | O22385^◘^ | | C | 4/64 | 114 | NITVNEAQSR | | | | 84 | 34 | | 17.7/5.0 (16.1/7.8) |
| 63* | E (21) | | Trypsin inhibitor | | | | | | | TC69848 | | S | 5/93 | 5.96e+003 |  | | | |  | 33 | | 22.6/4.6 (23.6/5.0) |
| 64 | L (21) | | Chalcone-flavone isomerase | | | | | | | TC69564 | | S | 7/122 | 3.31e+004 |  | | | |  | 30 | | 25.0/5.0 (24.5/5.9) |
| 66 | ↓ E (21) | | Aldehyde reductase | | | | | | | TC59970 | | S | 19/91 | 2.28e+009 |  | | | |  | 62 | | 36.7/6.0 (35.3/6.7) |
| 69* | ↑ E (21) | | Chitinase | | | | | | | TC68269 | | S | 11/200 | 2.83e+006 |  | | | |  | 40 | | 28.6/6.3 (34.9/7.4) |
| 75* | ↓ E (21) | | Beta VI allergen | | | | | | | TC68012 | | S | 8/174 | 3.18e+004 |  | | | |  | 50 | | 17.6/5.1 (18.0/5.3) |
| 97* | ↑ E (25) | | Thaumatin-like, pathogenesis related | | | | | | | TC59501 | | S | 5/150 | 4.8e+003 |  | | | |  | 22 | | 21.6/6.0 (26.8/5.9) |
| **Protein folding and turnover** | | | | | | | | | | | | | | | | | | | | | | |
| 7 | ↓ E (21) | | Cyclophilin  (Peptidyl-prolyl cis-trans isomerase)(PPIase) (Rotamase)  (*Ricinus communis*) | | | | | | | Q8VX73^◘^ | | C | 7/65 | 191 | | | FADENFIKK  ALCTGEKGVGR | | 60  74 | 36 | | 18.5/7.4 (18.4/8.9) |
| 8 | ↓ E (21) | | Cyclophilin (Peptidyl-prolyl cis-trans isomerase) (PPIase) (Rotamase)  (*Ricinus communis*) | | | | | | | Q8VX73^◘^ | | C | 8/65 | 216 | | | FADENFIKK  ALCTGEKGVGR | | 59  89 | 41 | | 18.5/8.0 (18.4/8.9) |
| 15 | L (25) | | Proteasome subunit alpha type-6  (*Glycine max*) | | | | | | | [O48551](http://www.ncbi.nlm.nih.gov/entrez/viewer.fcgi?db=protein&id=12229897)^◘^ | | C | 11/64 | 257 | | | TLVQQAR  NEAAEFR  AAGITSIGVR | | 47  50  56 | 39 | | 28.7/5.7  (27.5/5.8) |
| 104 | E (25) | | Cyclophilin (Peptydyl-propyl cis-trans isomerase) | | | | | | | MtD20401^◘^ | | S | 7/148 | 2.12e+004 | |  | | |  | 31 | | 17,3/7,6 (18.2/8.4) |
| **Transcription** | | | | | | | | | | | | | | | | | | | | | | |
| 21^■^ | ↑ E (25) | | Putative retroelement pol polyprotein  (*Arabidopsis thaliana*) | | | | | | | | Q9SK57^◘^ | C | 21/65 | 82 | | | |  |  | 14 | | 16.8/5.0 (185.0/8.8) |
| 22 | ↓ E (25) | | Eukaryotic translation initiation factor 5A-3 (eIF-5A-3)  (*Solanum tuberosum*) | | | | | | | | [P56335](http://www.ncbi.nlm.nih.gov/entrez/viewer.fcgi?db=protein&id=3024020)^◘^ | C | 4/53 | 108 | | | | TYPQQAGTIR  TYPQQAGTIRK | 55  25 | 7 | | 17.9/5.4 (17.7/5.8) |
| 23 | L (25) | | Reverse transcriptase-beet retrotransposon-related | | | | | | | | Q2HTR5^◘^ | C | 8/47 | 66 | | | |  |  | 24 | | 15.8/6.0  (30.5/8.9) |
| **Translation** | | | | | | | | | | | | | | | | | | | | | | |
| 3^■^ | ↓ E (21) | | Ribosomal protein small subunit 4 (fragment)  (*Saccoloina inaequale*) | | | | | | | | Q6SEK7^◘^ | C | 8/65 | 73 | | | |  |  | 47 | | 31.0/6.1 (19.9/10.3) |
| **Unknown function** | | | | | | | | | | | | | | | | | | | | | | |
| 84* | ↑ E (25) | | Unknown function | | | | | | | TC72846 | | S | 7/182 | 2.32e+003 | | | |  |  | 33 | | 27,6/6,3 (29.8/6.3) |
| **SA4087 infected/control** | | | | | | | | | | | | | | | | | | | | | | |
| **Defense and stress related** | | | | | | | | | | | | | | | | | | | | | | |
| 27* | ↓I (21) | | Chitinase | | TC106842 | | | | | | | C | 15/64 | 317 | | | | WSPSSADR  GPIQISWNYNYGQCGR  EQNPSSTYCQPSSEFPCASGK | 36  75  72 | 40 | | 33.3/6.9 (35.9/6.8) |
| 28* | ↓I (21) | | Chitinase | | TC106842 | | | | | | | C | 16/64 | 403 | | | | AFPNFANNGDTATK  GPIQISWNYNYGQCGR  EQNPSSTYCQPSSEFPCASGK | 73  80  99 | 42 | | 33.3/7.5 (35.9/6.8) |
| 35* | ↓I (25) | | Glycoside hydrolase, family 17 | | Q2HU16^◘^ | | | | | | | C | 10/52 | 160 | | | | IYLDNLIR  HFGVFYPNK  LYDPNQAALNALR | 31  21  32 | 25 | | 38.5/4.0 (40.0/6.6) |
| 39* | I (25) | | Chitinase | | TC106842 | | | | | | | C | 12/64 | 300 | | | | LYTYDAFISAAK  GPIQISWNYNYGQCGR  EQNPSSTYCQPSSEFPCASGK | 38  94  66 | 52 | | 21.9/6.1  (35.9/6.8) |
| 40* | ↓I (25) | | Chitinase | | TC106842 | | | | | | | C | 13/64 | 427 | | | | GPIQISWNYNYGQCGR  LPGYGTVTNIINGGLECGR  EQNPSSTYCQPSSEFPCASGK | 108  132  71 | 60 | | 19.2/6.1  (35.9/6.8) |
| 107 | I (21) | | Glutathione S-transferase | | TC59483 | | | | | | | S | 13/38 | 3.45e+004 | | | |  |  | 44 | | 25.5/5.9 (25.6/6.4) |
| 110* | ↑I (21) | | Glycin-rich RNA binding protein | | TC59317 | | | | | | | S | 7/59 | 4.17e+003 | | | |  |  | 39 | | 15.9/5.0 (18.7/6.5) |
| 114* | ↑I (21) | | Trypsin inhibitor | | TC62239 | | | | | | | S | 6/86 | 4.5e+003 | | | |  |  | 32 | | 19.3/6.0 (23.4/7.6) |
| **Metabolism** **(primary carbon metabolism)** | | | | | | | | | | | | | | | | | | | | | | |
| 26 | ↑I (21) | | Fructose-bisphosphate aldolase, cytoplasmic isozyme (*Cicer arietinum*) | | O65735^◘^ | | | | | | | C | 14/65 | 250 | GTVELAGTDGETTTQGLDGLGAR | | | | 136 | 36 | | 38.5/ 6.3 (38.6/6.2) |
| **Protein folding and turnover** | | | | | | | | | | | | | | | | | | | | | | |
| 41 | ↓I (25) | | Proteasome subunit alpha type 7  (*Cicer arietinum*) | | Q9SXU1^◘^ | | | | | | | C | 12/65 | 257 | KIVNLDDHIALACAGLK  AITVFSPDGHLFQVEYALEAVR | | | | 99  46 | 50 | | 19.0/6.3 (27.2/6.9) |
| **Cellular Processes and Signaling** | | | | | | | | | | | | | | | | | | | | | | |
| 36 | ↑I (25) | | Guanine nucleotide-binding protein subunit beta-like protein (*Medicago sativa*) | | O24076^◘^ | | | | | | | C | 11/65 | 278 | LWDLNAGTSAR  YTIQDGDAHSDWVSCVR | | | | 81  114 | 44 | | 38.5/7.4 (36.0/7.1) |
| 38 | ↓I (25) | | TH65-like protein (fragment) (*Lycopersicon esculentum*) | | | | Q949J3^◘^ | | | | | C | 13/65 | 74 |  | | | |  | 21 | | 26.8/6.1 (68.5/6.0) |
| 44^■^* | ↓I (25) | | Kinesin motor domain containing protein, expressed (*Oryza sativa*) | | | Q2QMU6^◘^ | | | | | | C | 28/67 | 93 |  | | | |  | 13 | | 18.0/4.7  (318.8/5.0) |
| **Translation** | | | | | | | | | | | | | | | | | | | | | | |
| 33^■^ | ↓I (21) | | TBP-binding protein-like  (*Oryza sativa*) | | Q8H2U2^◘^ | | | | | | | C | 9/67 | 67 |  | | | |  | 34 | | 18.8/5.4 (28.7/9.2) |
| **Unknown function** | | | | | | | | | | | | | | | | | | | | | | |
| 29^■^* | ↓I (21) | | Genomic DNA, chromosome 3, BAC clone:T19N8  (*Arabidopsis thaliana*) | | | | | Q9LH93^◘^ | | | | C | 11/65 | 68 |  | | | |  | 23 | | 31.0/6.1 (48.1/9.1) |
| **SA27774 infected/control** | | | | | | | | | | | | | | | | | | | | | | |
| **Defense and stress related** | | | | | | | | | | | | | | | | | | | | | | |
| 52* | ↑I (21) | | Proteinase inhibitor 20 | | | | [TC102534](http://compbio.dfci.harvard.edu/tgi/cgi-bin/tgi/tc_report.pl?tc=TC102534&species=Medicago) | | | | | C | 9/46 | 148 | | | | FDNDEAGR | 63 | 31 | | 23.2/6.5  (23.7/7.6) |
| 53* | ↑I (21) | | Proteinase inhibitor 20 | | | | [TC102534](http://compbio.dfci.harvard.edu/tgi/cgi-bin/tgi/tc_report.pl?tc=TC102534&species=Medicago) | | | | | C | 9/65 | 74 | | | |  |  | 31 | | 23.0/7.2  (23.7/7.6) |
| 54* | ↑I (21) | | Proteinase inhibitor 20 | | | | [TC102534](http://compbio.dfci.harvard.edu/tgi/cgi-bin/tgi/tc_report.pl?tc=TC102534&species=Medicago) | | | | | C | 9/65 | 74 | | | |  |  | 31 | | 22.6/7.6  (23.7/7.6) |
| 60 | ↓I (25) | | Glycine-rich RNA binding protein (*Medicago sativa*) | | | | Q9SP10^◘^ | | | | | C | 5/65 | 162 | | | | IINDRETGR  NITVNQAQSR | 34  82 | 56 | | 15.5/4.9  (10.8/4.5) |
| 127* | ↑I (21) | | Trypsin inhibitor | | | | TC69848 | | | | | S | 6/84 | 7.07e+004 | | | |  |  | 44 | | 21.2/4.5 (23.6/5.0) |
| 129 | ↑I (21) | | Trypsin inhibitor | | | | MtC00300^◘^ | | | | | S | 11/111 | 1.58e+006 | | | |  |  | 59 | | 17.8/4.8 (22.2/5.1) |
| 134* | ↑I (25) | | Trypsin inhibitor | | | | TC69291 | | | | | S | 6/69 | 1.25e+004 | | | |  |  | 33 | | 18.8/4.8 (25.4/5.3) |
| **Metabolism (primary carbon metabolism)** | | | | | | | | | | | | | | | | | | | | | | |
| 55^■^* | C (25) | | Putative transaldolase  (*Oryza sativa*) | | Q5JK10^◘^ | | | | | | | C | 13/66 | 76 |  | | | |  | 22 | | 66.9/6.6  (56.8/11.6) |
| **Transcription** | | | | | | | | | | | | | | | | | | | | | | |
| 51^■^ | ↑I (21) | | OSJNBa0056L23.24 (*Oryza sativa*) | | | | | Q7XL33^◘^ | | | | C | 17/60 | 69 | VTFDMLLEK | | | | 8 | 10 | | 21.7/4.9  (197.0/9.3) |
| 58^■^ | C (25) | | Copia-type reverse transcriptase-like protein  (*Arabidopsis thaliana*) | | | | | Q9M197^◘^ | | | | C | 18/60 | 78 |  | | | |  | 13 | | 66.2/7.5  (148.0/8.8) |
| **Unknown function** | | | | | | | | | | | | | | | | | | | | | | |
| 50^■^ | ↑I (21) | | Hypothetical protein OsI_034957  (*Oryza sativa*) | | | | | | A2ZEG4^◘^ | | | C | 8/65 | 89 |  | | | |  | 69 | | 22.2/4.9  (10.6/9.0) |
| 57 | C (25) | | Protein F22C12.6 (imported)  (*Arabidopsis thaliana*) | | | | | | Q9SH68^◘^ | | | C | 15/64 | 78 |  | | | |  | 21 | | 66.4/7.2  (88.8/8.8) |

^a^ Assigned spot numbers correspond to those indicated in additional files 2, 3, 4, 5, 6, 7, 8, 9, 10, 11, 12, 13.

^b^ Significant differences in volume (p ≤ 0.05) were considered. The ratio of the relative normalized volumes between genotypes and treatments was calculated. Symbols are as follows: E and L, indicate spot observed only in non-inoculated SA 27774 and SA 4087 genotype, respectively; ↑E and ↓E, mean spots more represented in SA 27774 and SA 4087 plants, respectively; C and I, indicate spot observed only in non-inoculated and inoculated plants, respectively; ↑I and ↓I, indicate spots most represented in inoculated plants and control, non-inoculated, respectively. Numbers in brackets correspond to days after inoculation when root tissues were sampled.

^c^ The PMF search was carried out on the Swiss-Prot and *M. truncatula* EST databases. When no correspondence was found in the Dana Faber database was indicated as (◘). When the identified protein is from an organism different from *M. truncatula*, the plant species is indicated in brackets.

^d^ Staining procedure: C (Coomassie), S (silver).

^e^ Number of matched peptides with respect to the total with PMF data.

^f^ Fragmented peptides sequence (up to three peptides were selected to MSMS fragmentation)

^g^ Aminoacid sequence coverage of the identified proteins.

^h^ Experimental and theoretical mass (*Mr*, kDa) and p*I* of identified proteins. Experimental values were calculated with PD-Quest software (BioRad) and standard molecular mass markers. Theoretical values were retrieved from the protein database.

^■^ Protein spots that do not comply with all identification confidence parameters.

* Presence of an N-terminal signal sequence using SignalP 3.0 (CBS): http://www.cbs.dtu.dk/services/SignalP
