# Supplementary material for: Differential expression proteomics to investigate responses and resistance to Orobanche crenata in Medicago truncatula
Source: BMC Genomics. 2009 Jul 3;10:294. doi: 10.1186/1471-2164-10-294 (PMC2714000; doi:10.1186/1471-2164-10-294)
Supplement: Additional file 16 — Root growth of Medicago truncatula SA 4087 and SA 27774 control non-inoculated and inoculated plants. [file 1471-2164-10-294-S16.doc]

Root growth of *Medicago truncatula* SA 4087 and SA 27774 control non-inoculated and infected plants

| **Accession** | **Fresh weight (g/plant) ^a^** | |
| --- | --- | --- |
|  | **No inoculated** | **Inoculated** |
| SA 4087 | 0.48 | 0.24 |
| SA 27774 | 0.22 | 0.24 |

a Fresh weight of roots from both genotypes in non-inoculated and inoculated plants was monitored 25 dpi. No statistically significant differences were observed (LSD, P<0.05)
